# Supplementary material for: Slowly Reducible Genetically Encoded Green Fluorescent Indicator for In Vivo and Ex Vivo Visualization of Hydrogen Peroxide
Source: Int J Mol Sci. 2019 Jun 27;20(13):3138. doi: 10.3390/ijms20133138 (PMC6650888; doi:10.3390/ijms20133138)
Supplement: Supplementary file 1 [file ijms-20-03138-s001.zip › SupplementaryInformation/Subach et al 2019 Supplementary FS.pdf]

Supplementary Information

Slowly Reducible Genetically Encoded Green Fluorescent Indicator for In Vivo and Ex Vivo Visualization of Hydrogen Peroxide

Oksana M. Subach, Tatiana A. Kunitsyna, Olga A. Mineyeva, Alexander A. Lazutkin, Dmitri V. Bezryadnov, Natalia V. Barykina, Kiryl D. Piatkevich, Yulia G. Ermakova, Dmitry S. Bilan, Vsevolod V. Belousov, Konstantin V. Anokhin, Grigori N. Enikolopov, and Fedor V. Subach

Supplementary Figures, Video, Tables, Materials and Methods, Results, and References.

Figure S1. 3
Figure S2. 4
Figure S3. 5
Figure S4. 6
Figure S5. 7
Figure S6. 8
Figure S7. 9
Figure S8. 10
Figure S9. 11
Figure S10. 12
Figure S11. 13
Figure S12. 14
Figure S13. 15
Figure S14. 16
Figure S15. 17
Figure S16. 18
Figure S17. 19
Figure S18. 20
Figure S19. 21
Figure S20. 22
Figure S21. 23
Figure S22. 24
Figure S23. 25
Figure S24. 26
Figure S25. 27
Figure S26. 28
Figure S27. 29

Video S1. 30
Video S2. 31

Table S1. 32
Table S2. 33
Table S3. 34
Table S4. 35
Table S5. 36

Table S6. 37

Scheme 1 38

Materials and Methods 39-42

Results 42-45

References 46

```
                       10        20        30        40        50
                        |         |         |         |         |
GFP             MSKGEELFTGVVPILVELDGDVNGHKFSVSGEGEGDATYGKLTLKFICTTG---KLPVPWP
EYFP           MVSKGEELFTGVVPILVELDGDVNGHKFSVSGEGEGDATYGKLTLKFICTTG---KLPVPWP
TurboGFP          MESDESGLPAMEIECRITGTLNGVEFELVGGGEGTPEQGRMTNKMKSTKG---ALTFSPY
mNeonGreen   MVSKGEEDNMASLPATHELHIFGSINGVDFDMVGQGTGNPNDGYEELNLKSTKG---DLQFSPW

                60        70        80        90         100       110
                 |    *** |         |         |         |         |
GFP             TLVTTFSYGVQCFSRYPDHMKQHDFFKSAMPEGYVQERTIFFKDDGNYKTRAEVKFEGDT
EYFP            TLVTTFGYGLQCFARYPDHMKQHDFFKSAMPEGYVQERTIFFKDDGNYKTRAEVKFEGDT
TurboGFP        LLSHVMGYGFYHFGTYPSGYE-NPFLHAINNGGYTNTRIEKYEDGGVLHVSFSYRYEAGR
mNeonGreen      ILVPHIGYGFHQYLPYPDGMS-PFQAAMVDGSGYQVHRTMQFEDGASLTVNYRYTYEGSH

               120       130       140       150       160       170
                 |         |         |         |         |         |
GFP             LVNRIELKGIDFKEDGNILGHKLEYNYNSHNVYIMADKQKNGIKVNFKIRHNIEDGSVQL
EYFP            LVNRIELKGIDFKEDGNILGHKLEYNYNSHNVYIMADKQKNGIKVNFKIRHNIEDGSVQL
TurboGFP        VIGDFKVMGTGFPEDSVIFTDKII-RSNATVEHLHPM-GDNDLDGSFTRTFSLRDGGYYS
mNeonGreen      IKGEAQVKGTGFPADGPVMTNSLT-AADWCRSKKTYP-NDKTIISTFKWSYTTGNGKRYR

             180       190          200       210       220       230
               |         |            |         |         |         |
GFP            ADHYQQNTPIGDG---PVLLPDNHYLSTQSALSKDPNEKRDHMVLLEFVTAAGITHGMDELYK
EYFP           ADHYQQNTPIGDG---PVLLPDNHYLSYQSALSKDPNEKRDHMVLLEFVTAAGITLGMDELYK
TurboGFP       SVVDSHMHFKSAIHPSILQNGGPMFAFRRVEEDHS----NTELGIVEYQHAFKTPDADAGEE
mNeonGreen     STARTTYTFAKPMAANYLKNQP-MYVFRKTELKHS----KTELNFKEWQKAFTDVMGMDELYK
```

Figure S1. Alignment of the amino acid sequences of mNeonGreen with GFP, EYFP and TurboGFP. Alignment numbering follows that of *Aequorea victoria* GFP. Residues buried in β-can are shaded. Stars indicate residues that are forming chromophore. Sites of permutation in mNeonGreen and EYFP are indicated with green arrow.

```
          80        90       100       110       120       130
           |         |         |         |         |         |
Library   MEMASQQGETMSGPLHIGLIPTVGPYLLPHIIPMLHQTFPKLEMYLHEAQTHQLLAQLDS
NeonOxIrr MEMASQQGETMSGPLHIGLISTVGPYLLPHIIPMLHQTFPKLEMYLHEAQTHQLLAQLDS

         140       150       160       170       180       190
           |         |         |         |         |         |
Library   GKLDCVILALVKESEAFIEVPLFDEPMLLAIYEDHPWANRECVPMADLAGEKLLMLEDGH
NeonOxIrr GKLDCVILAPVKESEAFIEVPLFDEPMLLAIYEDHPWANRECVPMADLAGEKLLMLEDGH

         200  205 145  150       160       170       180       190
          *|    |   |    |         |         |         |         |
Library   CLRDQAMXXXADWCRSKKTYP-NDKTIISTFKWSYTTGNGKRYRSTARTTYTFAKPMAANY
NeonOxIrr CLRDQAMAYFADWCRSKKTYP-NDKTIISTFKWSYTTGNGKRYRSTARTTYTFAKPMAANY

               200       210       220       230     238            1
                 |         |         |         |       |            |
Library   LKNQP-MYVFRKTELKHS----KTELNFKEWQKAFTDVMGMDELYKVDGGSGGTGMVSKGEEDNM
NeonOxIrr LKNQP-MYVVRKTELKHS----KTELNFKEWQKAFTDVMGMDELYKVDGGSGGTGMASKGEEDNM

           10        20        30        40        50          60
            |         |         |         |         |            |    ***
Library   ASLPATHELHIFGSINGVDFDMVGQGTGNPNDGYEELNLKSTKG---DLQFSPWILVPHIGYG
NeonOxIrr ASLPATHELHIFGSINGADFDMVGQGTGNPIDGYEELNLKSTKG---DLQFSPWILVPHIGYG

           70        80        90       100       110       120
            |         |         |         |         |         |
Library   FHQYLPYPDGMS-PFQAAMVDGSGYQVHRTMQFEDGASLTVNYRYTYEGSHIKGEAQVKGT
NeonOxIrr FHQYLPYPDGMS-PFQAAMVDGSGYQVHRTMQFEDGASLTVNYRYTYEGSHIKGEAQVKGT

         130       140 144 206 210       220        230       240
           |         |   |   | * |         |          |         |
Library   GFPADGPVMTNSLT-AXXXGFCFEAGADEDTHFRAT-SLETLRNMVAAGSGITLLPALAVPP
NeonOxIrr GFPADGPVMTNSLT-AIDNGFCFEAGADEDTHFRAT-SLETLRNMVAAGSGITLLPALAVPP

          250       260        270       280       290       300
            |         |          |         |         |         |
Library   ERKRDGVVYLPCIKPE-PRRTIGLVYRPGSPLRSRYEQLAEAIRARMDGHFDKVLKQAV
NeonOxIrr ERKRDGVVYLPCTKPE-PRRTIGLVYRPGSPLRSRYEQLAEAIRARMDGHFDKVLKQAV
```

Figure S2. Alignment of the amino acid sequences of original library with NeonOxIrr. Alignment numbering for fluorescent part is shown in green color and follows that of avGFP. Residues from fluorescent part buried in β-can are highlighted with green. Amino acids located in linker connecting N- and C-terminal ends of fluorescent protein are selected with yellow color. Green stars indicate residues that are forming chromophore. Alignment numbering for sensory part is shown in black and follows that of OxyR from *E.Coli*. Conserved amino acids from sensory part are shaded. Two conserved cysteine residues (C199 and C208) that form disulfide bond are marked with black asterisks.

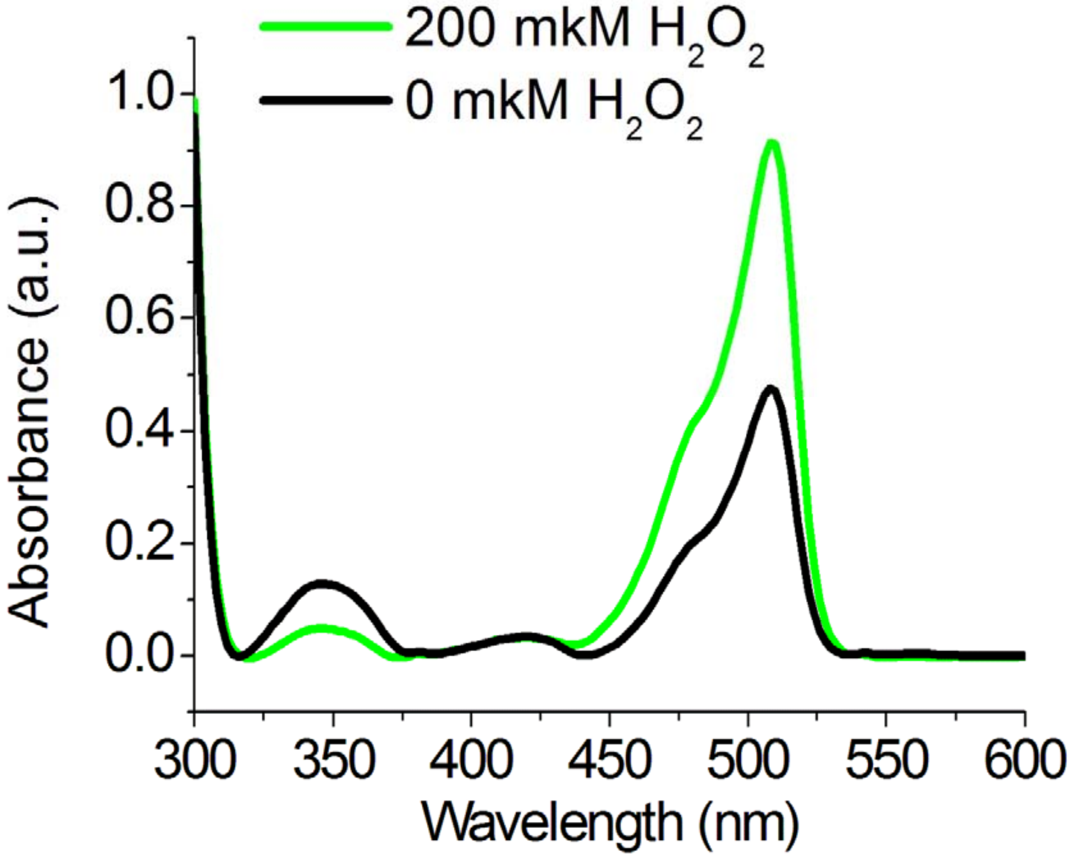

Figure S3. Absorbance spectra of the NeonOxIrr indicator in the presence and in the absence of H_2O_2.

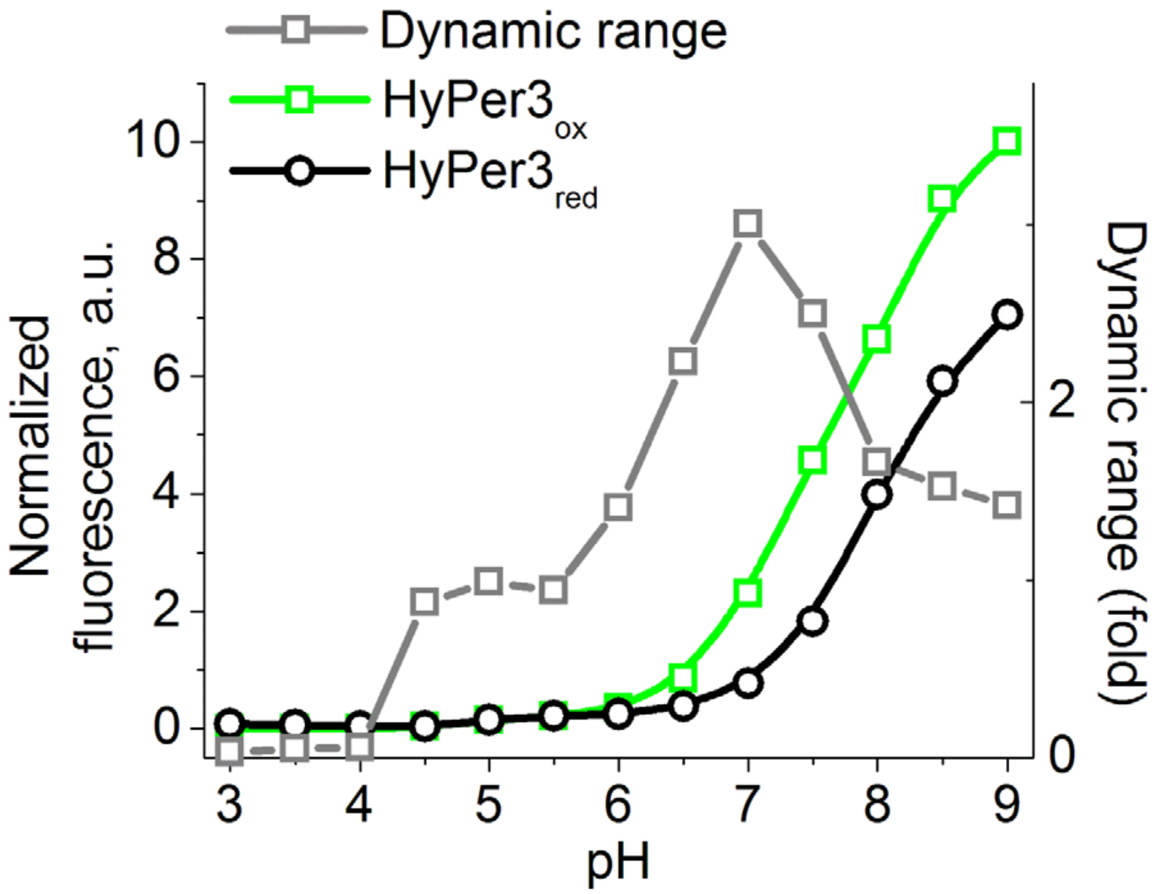

Figure S4. pH dependence of fluorescence and dynamic range for HyPer3 indicator. Equilibrium pH dependences for dynamic range and the fluorescence of HyPer3 in the oxidized and reduced (in the presence of 14 mM DTT) states.

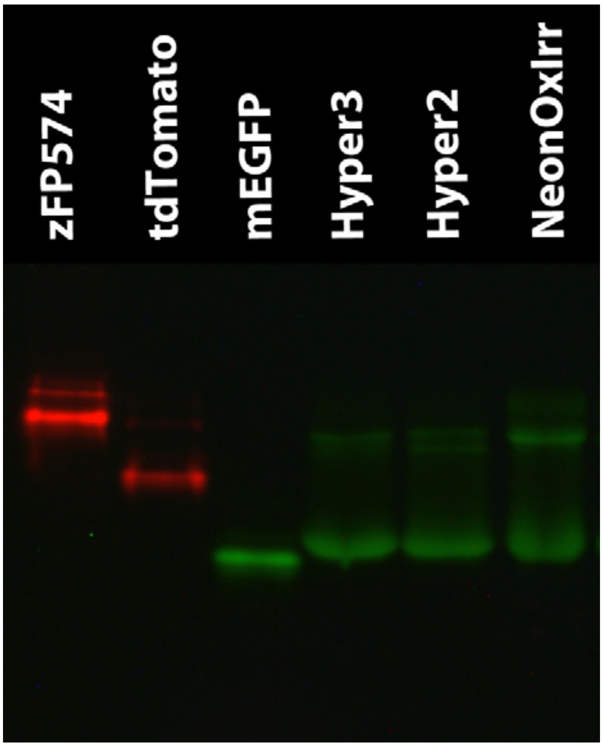

Figure S5. Semi-native polyacrylamide gel with redox sensors. 10 μg of the freshly purified fluorescent proteins were applied without heating in 10 μl aliquots onto the 15% polyacrylamide gel containing 0.01% SDS. The gel was run using a low voltage at +4°C. mEGFP[1], tdTomato[2] and zFP574 (Ref. [3]) were applied as monomeric, dimeric and tetrameric native protein standards, respectively. The gel was photographed using a Leica M205FA fluorescence stereomicroscope. Green fluorescence was registered by 480/40BP excitation and 540/40BP emission filters, respectively. Red fluorescence was registered by 540/40BP excitation and 620/60BP emission filters, respectively.

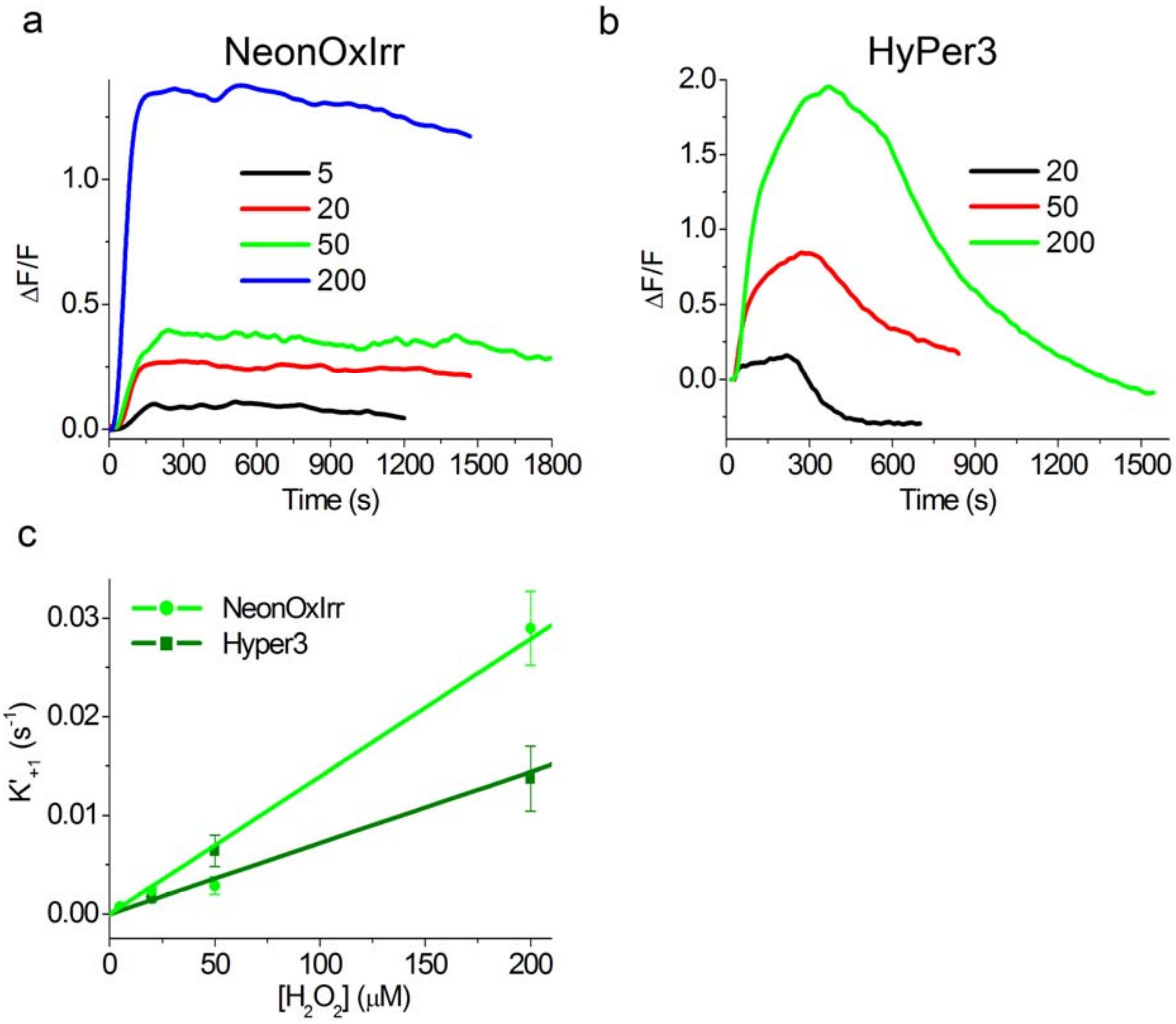

Figure S6. Comparison of the oxidation rate constants for the NeonOxIrr and HyPer3 indicators in the cytoplasm of HeLa cells. (a, b) Time dependence of ΔF/F response of NeonOxIrr **(a)** and HyPer3 **(b)**, averaged across 5-10 cells. Final concentrations of external H_2O_2 are shown in μM. (**c**) K_{+1}' values (in sec^{-1}) at each H_2O_2 concentration were calculated as initial slope on the linear part of $\ln[(F_{max}-F_{min})/(F_{max}-F(t))]$ vs time dependence, where F_{max}, F_{min} and F(t) are maximal, minimal and at given time fluorescence intensities values, respectively. k_{+1} oxidation rates (in $M^{-1}sec^{-1}$) were calculated as a slope of K_{+1}' vs H_2O_2 concentration dependence (please, see ESI Table S3).

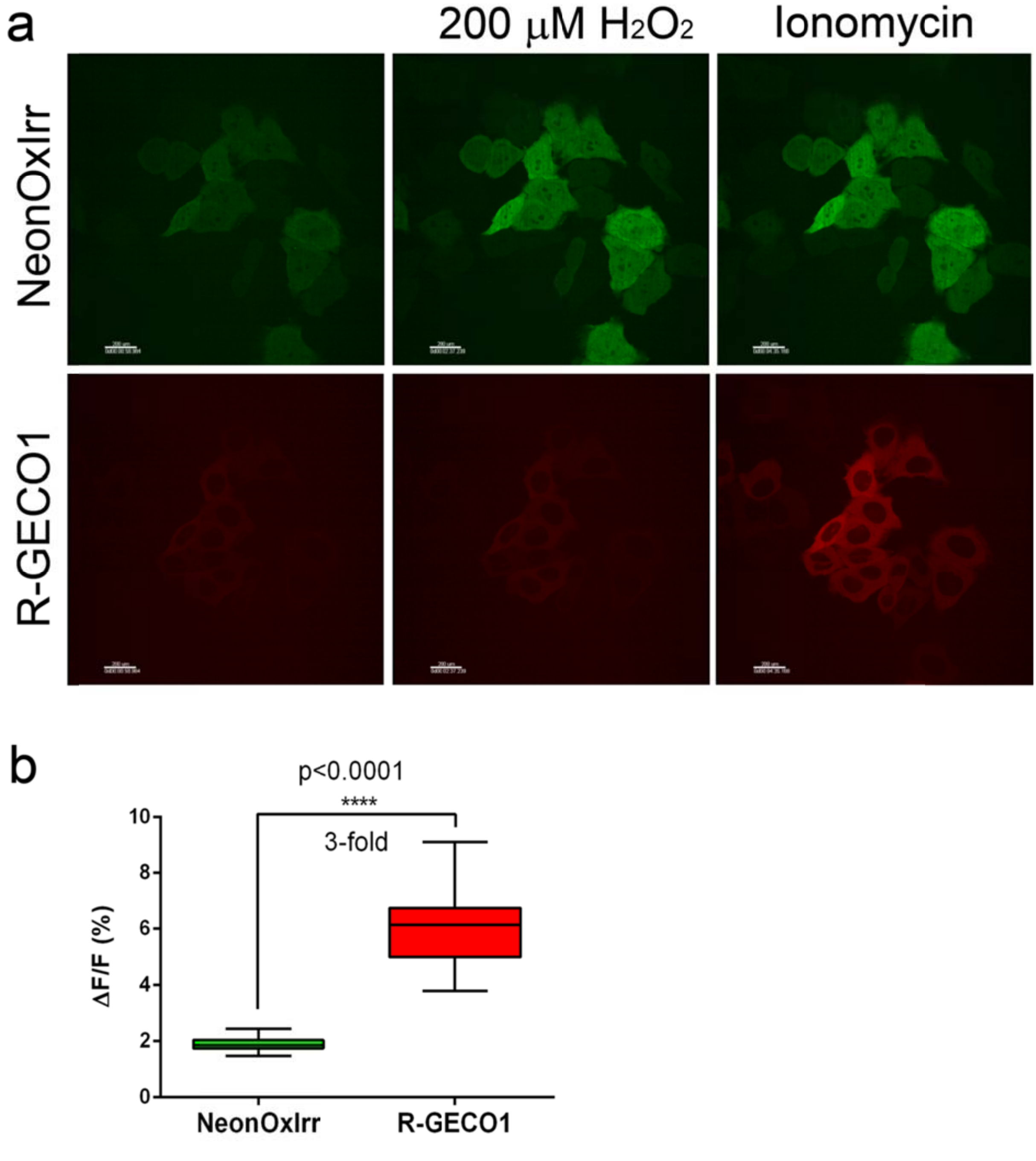

Figure S7. Simultaneous two-color imaging of H_2O_2 and calcium transients in HeLa cells. Expanded data to Figure 2d in the Main text. (**a**) Confocal images of the HeLa cells before and upon addition of 200 μM H_2O_2 and 2.5 μM Ionomycin. Scale bars are shown as white bands. (**b**) ΔF/F responses averaged across 19 cells in two cultures. The box extends from the 25th to 75th percentiles. The line in the middle of the box is plotted at the median. The whiskers go down to the smallest value and up to the largest.

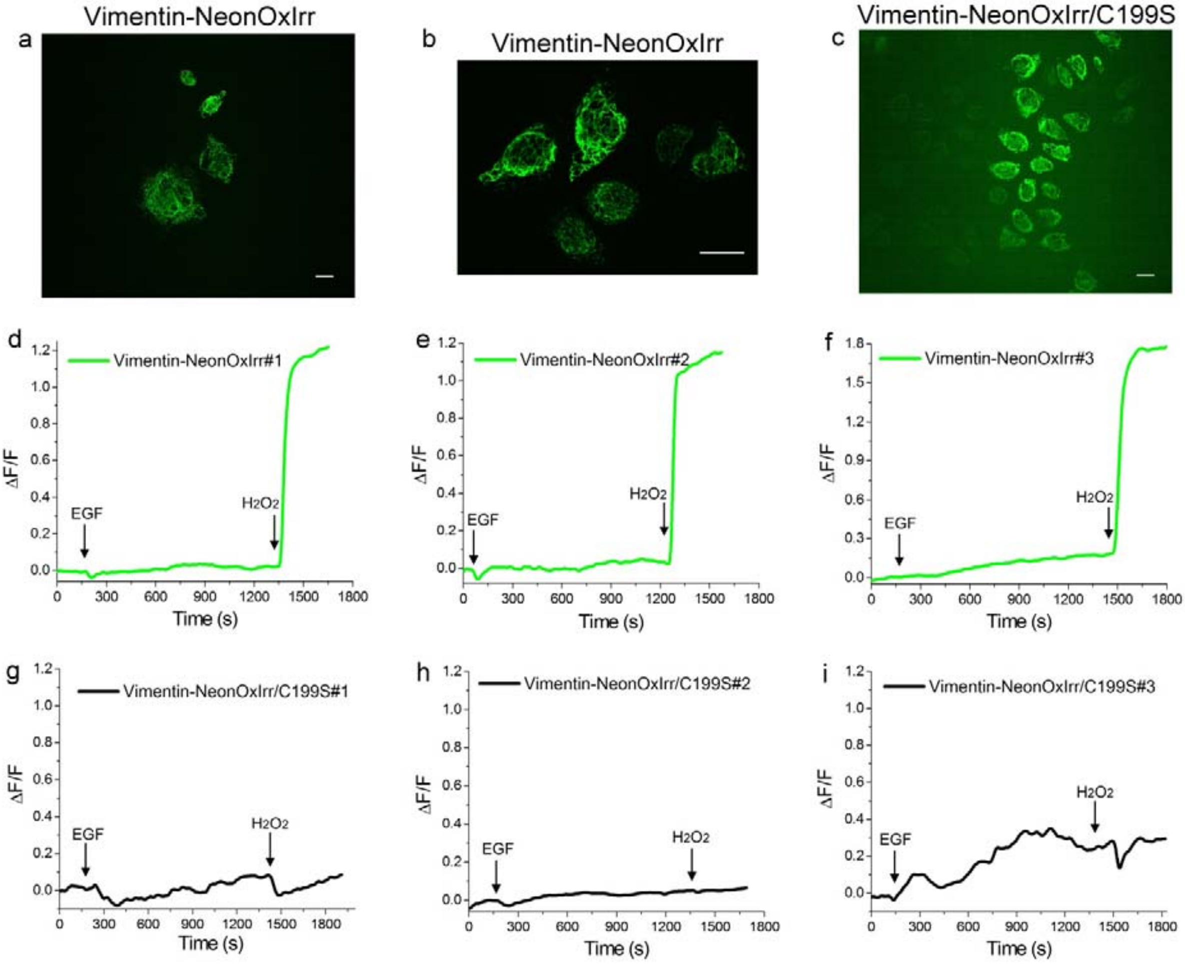

Figure S8. Response of vimentin-NeonOxIrr fusion protein to external H_2O_2 and EGF-induced endogenous H_2O_2 production in the cytoplasm of live HeLa Kyoto cells. (a) Example of confocal image of HeLa Kyoto cells expressing vimentin-NeonOxIrr fusion protein. (**b**) Similar to (a) but with enlarged cell size to look at vimentin filaments. (**c**) Example of confocal image of HeLa Kyoto cells expressing vimentin-NeonOxIrr/C199S fusion protein. (**d-f**) Dynamics of green fluorescence response of the vimentin-NeonOxIrr indicator to the consecutive addition of EGF (100ng/ml) and H_2O_2 (200 μM). Three panels present dependences of averaged fluorescence responses for cells inside three different cell cultures. **(g-i)** Dynamics of green fluorescence response of the vimentin-NeonOxIrr/C199S indicator (with blocked sensitivity to H_2O_2) to the consecutive addition of EGF (100ng/ml) and H_2O_2 (200 μM). Three panels present dependences of averaged fluorescence responses for cells inside three different cell cultures. Times of the addition of EGF and H_2O_2 are shown with arrows. Dynamics of fluorescence changes were extracted from series of images acquired on confocal microscope. Scale bars – 200 (**a, c**) and 100 (**b**) μm.

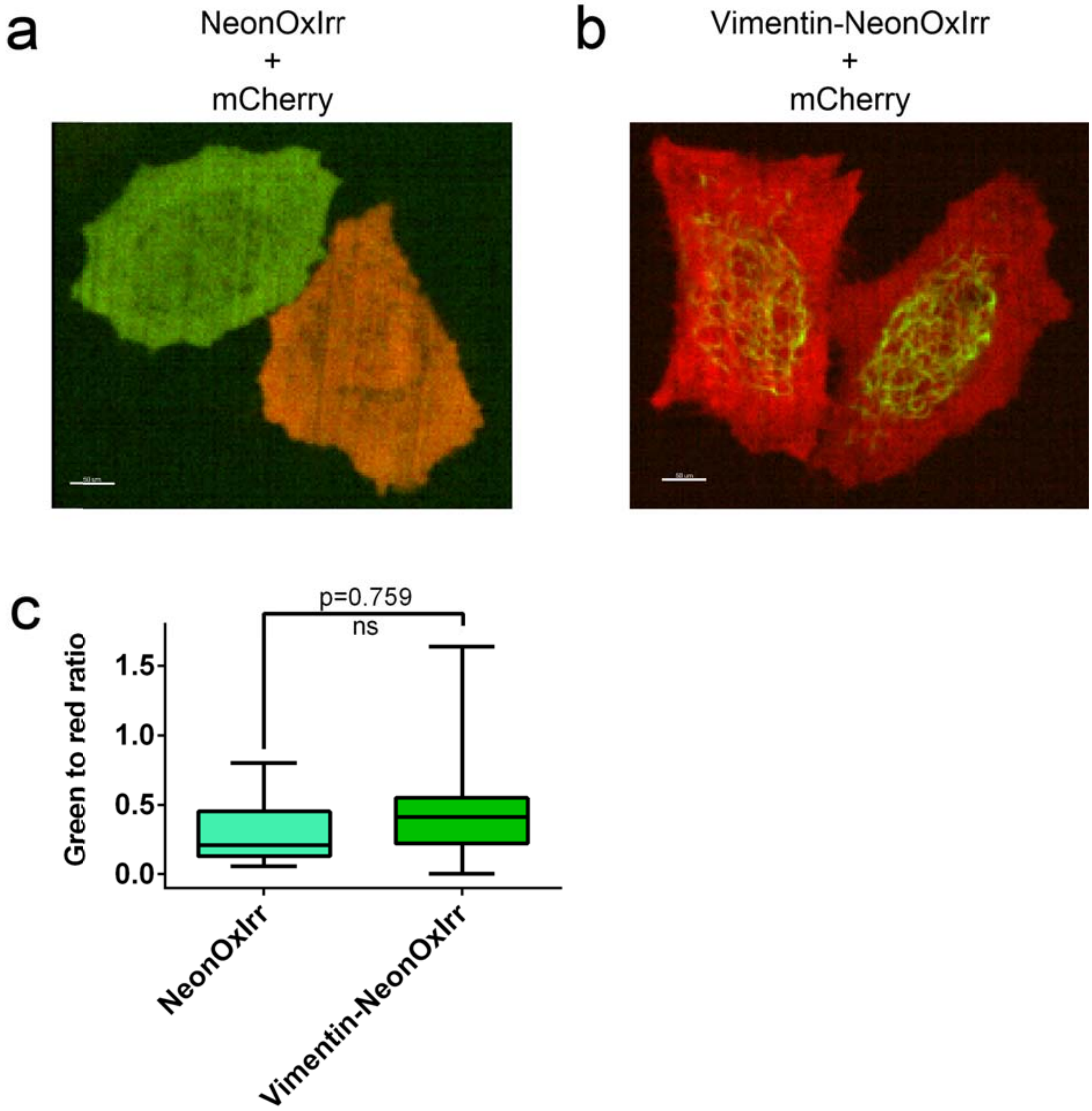

Figure S9. Comparison of expression levels of NeonOxIrr and vimentin-NeonOxIrr fusion in HeLa cells. The NeonOxIrr (**a**) and vimentin-NeonOxIrr (**b**) were transiently co-expressed with mCherry protein in HeLa Kyoto cells and oxidized with 200 μM H_2O_2 5-10 min before imaging. Confocal images of cells in overplayed green and red channels are shown. Scale bar – 50 μm. (**c**) Averaged green fluorescence mean intensities values normalized to the mean values of red fluorescence of mChery protein (green to red ratio) were practically the same.

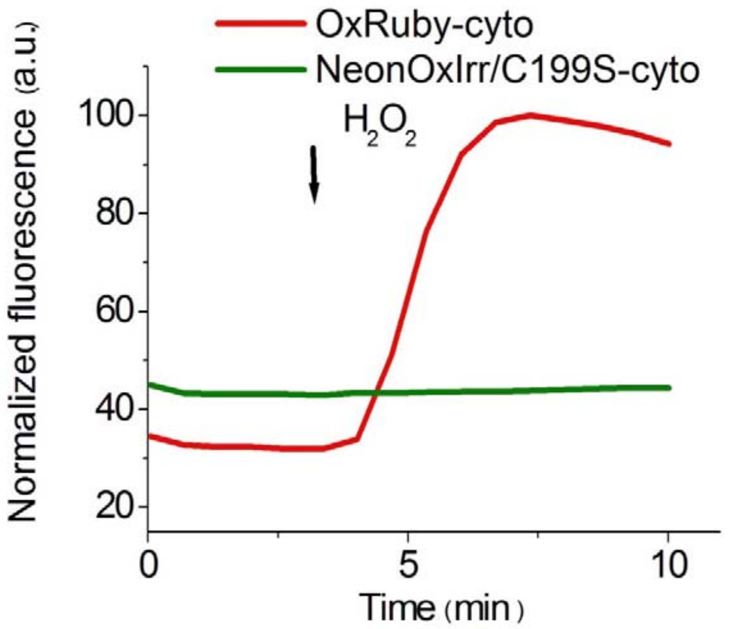

Figure S10. Inhibition of reaction of NeonOxIrr carrying Cys199Ser mutation to external H_2O_2 when expressed in cytoplasm of HeLa cells. NeonOxIrr carrying Cys199Ser mutation was expressed in cytoplasm of live HeLa Kyoto cells. Addition of external H_2O_2 till final concentration of 200 μM is shown with arrow. OxRuby red sensor (our unpublished red indicator for H_2O_2) was co-expressed in the cytoplasm of cells to confirm the presence of H_2O_2.

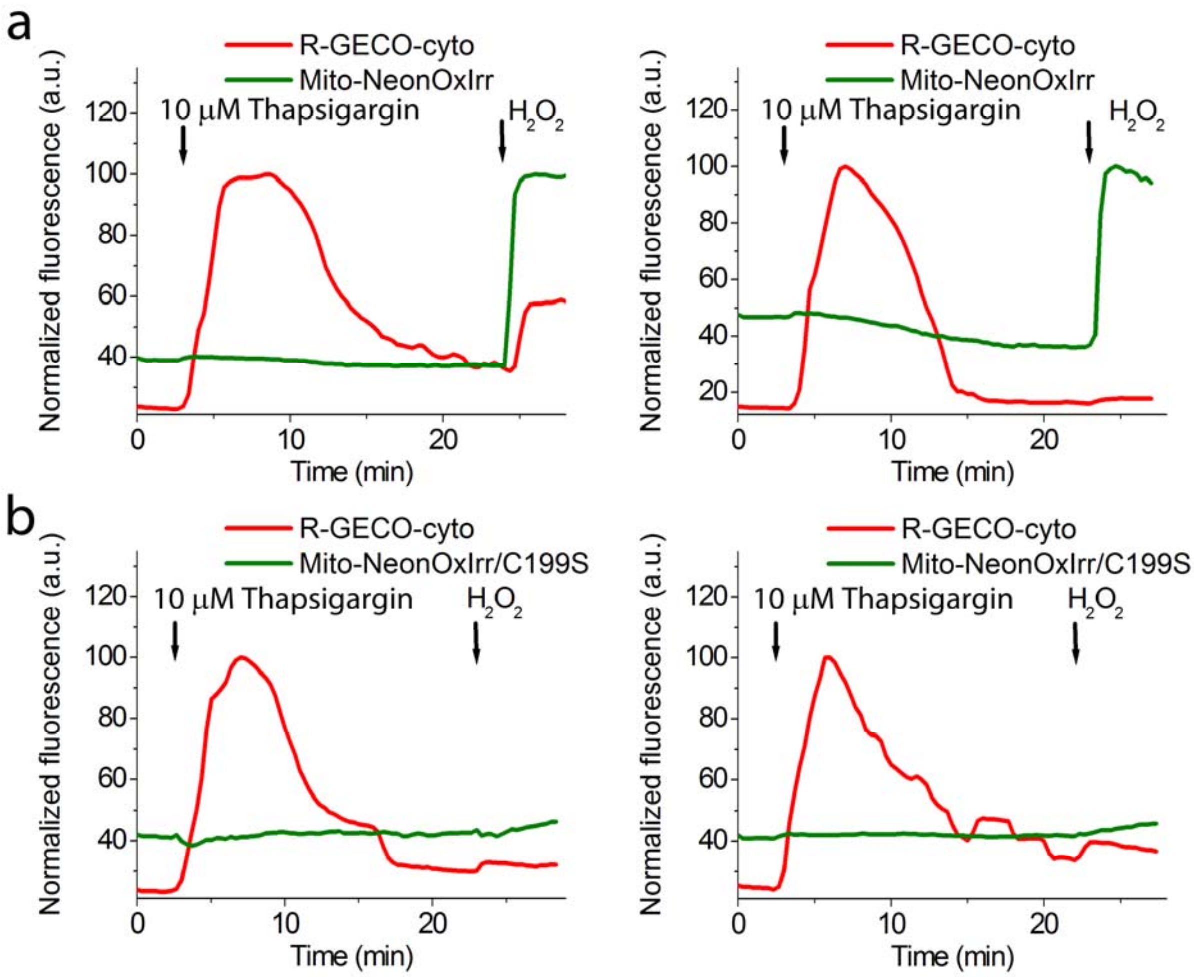

Figure S11. Response of NeonOxIrr to thapsigargin-induced endogenous H_2O_2 production in the lumen of mitochondria of live HeLa cells. (a) Dynamics of green fluorescence of the NeonOxIrr indicator to the consecutive addition of thapsigargin (10 μM) and H_2O_2 (200 μM). **(b)** Dynamics of green fluorescence of the NeonOxIrr/C199S indicator (with blocked sensitivity to H_2O_2) to the consecutive addition of thapsigargin (10 μM) and H_2O_2 (200 μM). Times of the addition of thapsigargin and H_2O_2 are shown with arrows. Fluorescence of red calcium indicator R-GECO1 co-expressed in the same cells was registered in red channel simultaneously with green fluorescence. Dynamics of fluorescence changes were extracted from series of images acquired on confocal microscope. Fluorescence was normalized to the 100% maximal fluorescence achieved for NeonOxIrr after the H_2O_2 addition. Left and right panels correspond to different cell cultures.

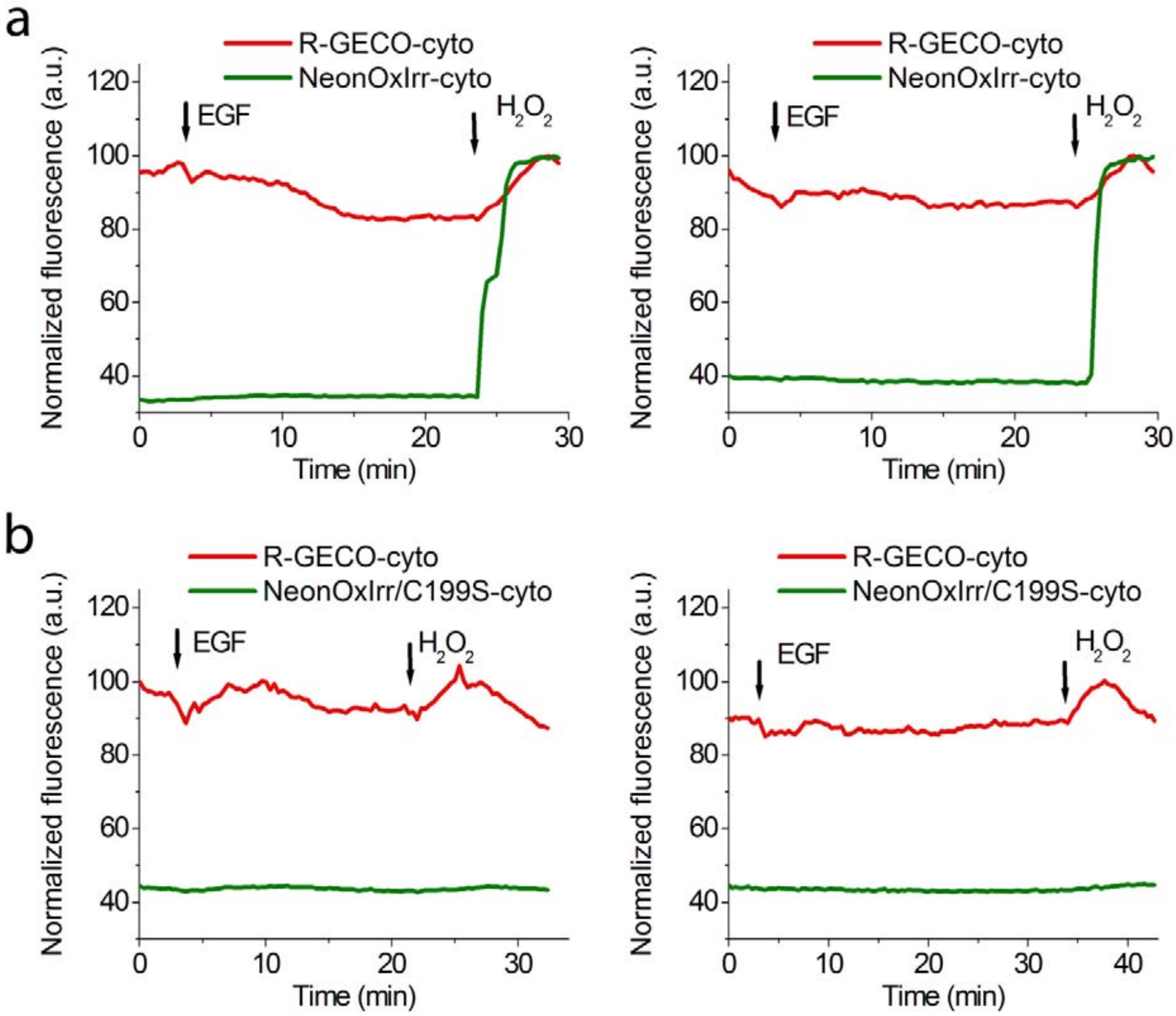

Figure S12. Response of NeonOxIrr to EGF-induced endogenous H_2O_2 production in the cytoplasm of live HeLa cells. (a) Dynamics of green fluorescence of the NeonOxIrr indicator to the consecutive addition of EGF (100ng/ml) and H_2O_2 (200 μM). **(b)** Dynamics of green fluorescence of the NeonOxIrr/C199S indicator (with blocked sensitivity to H_2O_2) to the consecutive addition of EGF (100ng/ml) and H_2O_2 (200 μM). Times of the addition of EGF and H_2O_2 are shown with arrows. Fluorescence of red calcium indicator R-GECO1 co-expressed in the same cells was also registered in red channel simultaneously with green fluorescence. Dynamics of fluorescence changes were extracted from series of images acquired on confocal microscope. Fluorescence was normalized to the 100% maximal fluorescence achieved for NeonOxIrr after the H_2O_2 addition. Left and right panels correspond to different cell cultures.

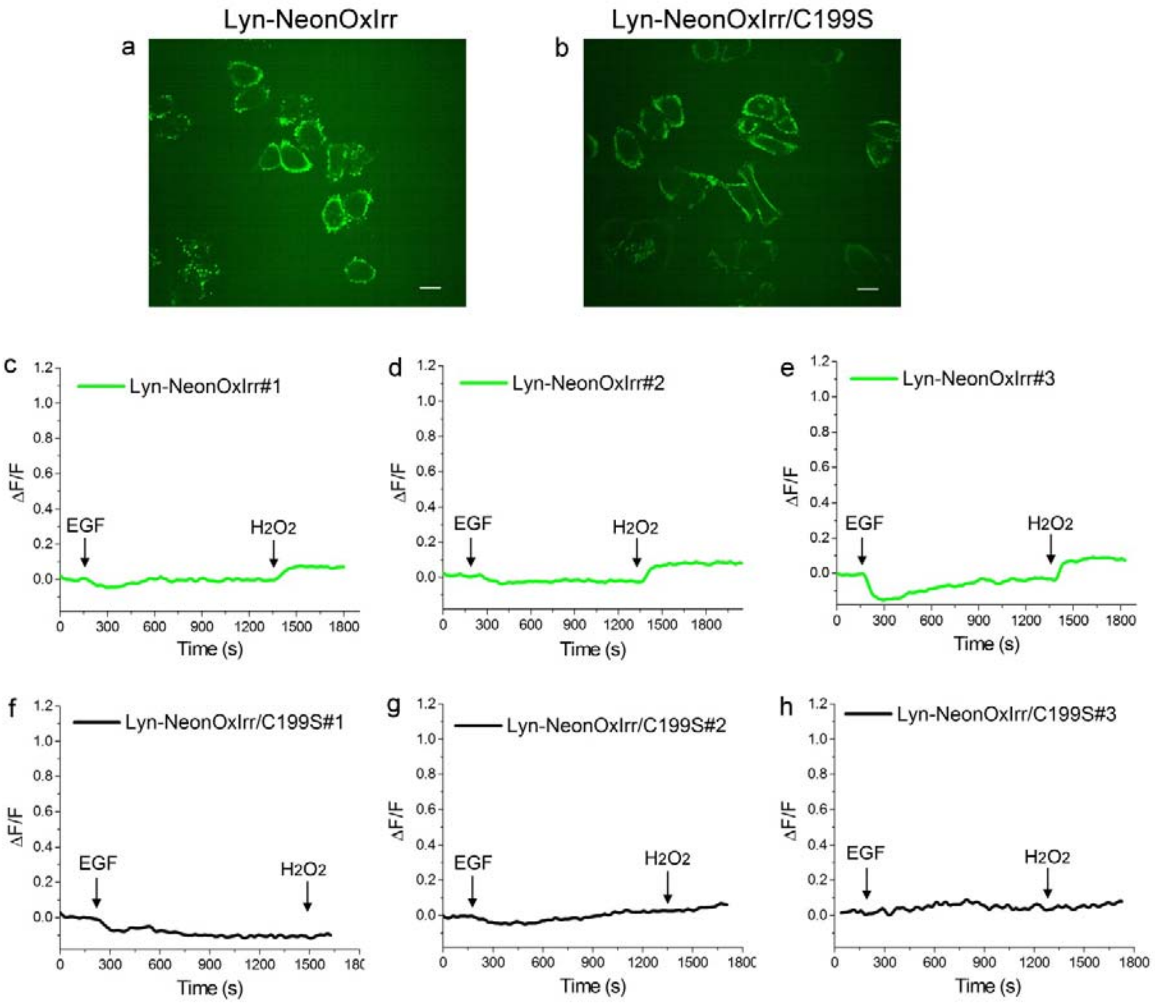

Figure S13. Response of Lyn-NeonOxIrr fusion protein to EGF-induced endogenous H_2O_2 production at the plasma membrane of live HeLa Kyoto cells. (a) Example of confocal image of HeLa Kyoto cells expressing Lyn-NeonOxIrr fusion protein. (**b**) Example of confocal image of HeLa Kyoto cells expressing Lyn-NeonOxIrr/C199S fusion protein. (**c-e**) Dynamics of green fluorescence response of the Lyn-NeonOxIrr indicator to the consecutive addition of EGF (100ng/ml) and H_2O_2 (200 μM). Three panels present dependences of averaged fluorescence responses for cells inside three different cell cultures. **(f-h)** Dynamics of green fluorescence response of the Lyn-NeonOxIrr/C199S indicator (with blocked sensitivity to H_2O_2) to the consecutive addition of EGF (100ng/ml) and H_2O_2 (200 μM). Three panels present dependences of averaged fluorescence responses for cells inside three different cell cultures. Times of the addition of EGF and H_2O_2 are shown with arrows. Dynamics of fluorescence changes were extracted from series of images acquired on confocal microscope. Scale bars – 200 μm.

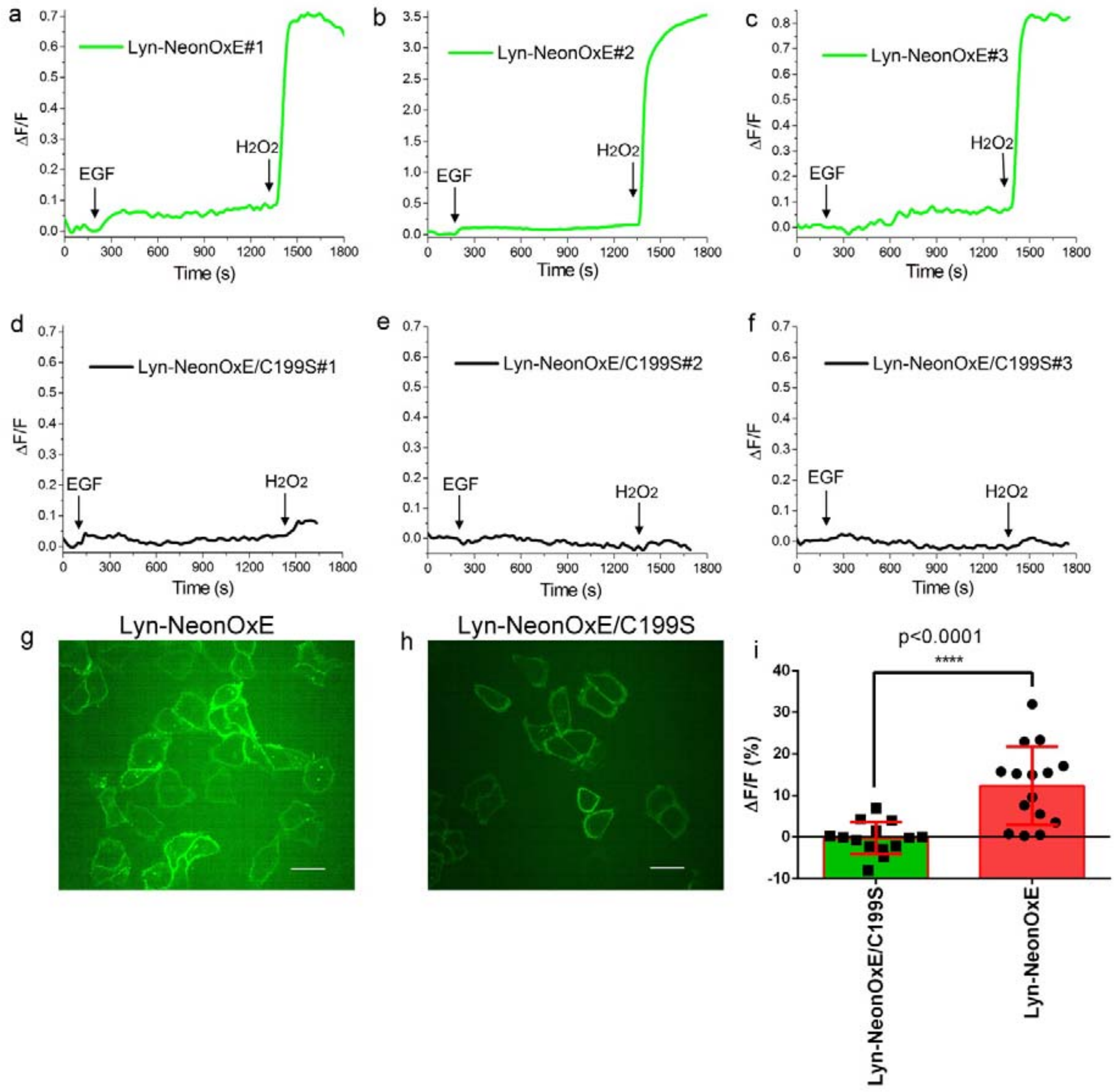

Figure S14. Response of Lyn-NeonOxE fusion protein to EGF-induced endogenous H_2O_2 production at the plasma membrane of live HeLa Kyoto cells. (**a-c**) Dynamics of green fluorescence response of the Lyn-NeonOxE indicator to the consecutive addition of EGF (100ng/ml) and H_2O_2 (200 µM). Three panels present dependences of averaged fluorescence responses for cells inside three different cell cultures. **(d-f)** Dynamics of green fluorescence response of the Lyn-NeonOxE/C199S indicator (with blocked sensitivity to H_2O_2) to the consecutive addition of EGF (100ng/ml) and H_2O_2 (200 µM). Three panels present dependences of averaged fluorescence responses for cells inside three different cell cultures. Times of the addition of EGF and H_2O_2 are shown with arrows. Dynamics of fluorescence changes were extracted from series of images acquired on confocal microscope. **(g)** Example of confocal image of HeLa Kyoto cells expressing Lyn-NeonOxE fusion protein. (**h**) Example of confocal image of HeLa Kyoto cells expressing Lyn-NeonOxE/C199S fusion protein. (**i**) Fluorescence responses ΔF/F (%) for Lyn-NeonOxE and Lyn-NeonOxE/C199S at the plasma membrane of HeLa Kyoto cells after addition of EGF. Scale bars – 300 µm.

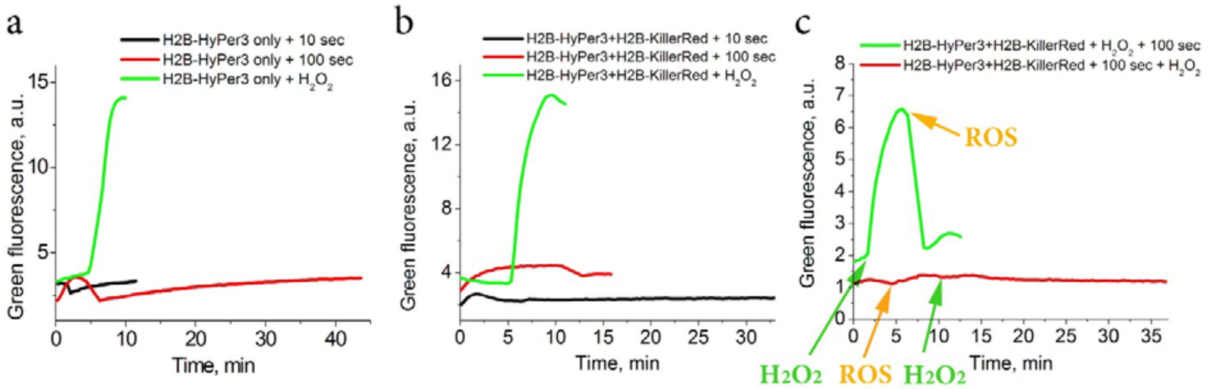

Figure S15. Reaction of green HyPer3 redox sensor with optogenetic ROS generated by KillerRed red fluorescent protein that is co-expressed with sensor in nucleus of live HEK293T mammalian cells. Response of H2B-HyPer3 sensor expressed in the nucleus of live HEK293T cells along (**a**) or co-expressed with H2B-KillerRed fluorescent protein (**b, c)** to H_2O_2 and yellow light (545/30 nm excitation light power of 3.1 mW/cm2 before 60x objective lens) illumination for 10 and 100 sec. (**a, b**) Black and red plots illustrate changes of fluorescence of H2B-HyPer3 in nucleus upon its illumination with yellow light for 10 and 100 sec, respectively. Green graphs show reactions of sensor to addition of external H_2O_2 (200 μM final concentration). (**c**) Green curve illustrates changes of fluorescence of H2B-HyPer3 in nucleus upon addition of 200 μM H_2O_2 followed by illumination with yellow light for 100 sec at peak of fluorescence maxima corresponding to maximally oxidized H2B-HyPer3. Red curve illustrates changes of fluorescence of H2B-HyPer3 in nucleus upon illumination with yellow light for 100 sec followed by addition of 200 μM H_2O_2. Additions of H_2O_2 and irradiation with yellow light are shown with green and yellow arrows, respectively. The time dependences of green fluorescence were extracted from time-lapse series of fluorescent confocal images of HEK293T cells.

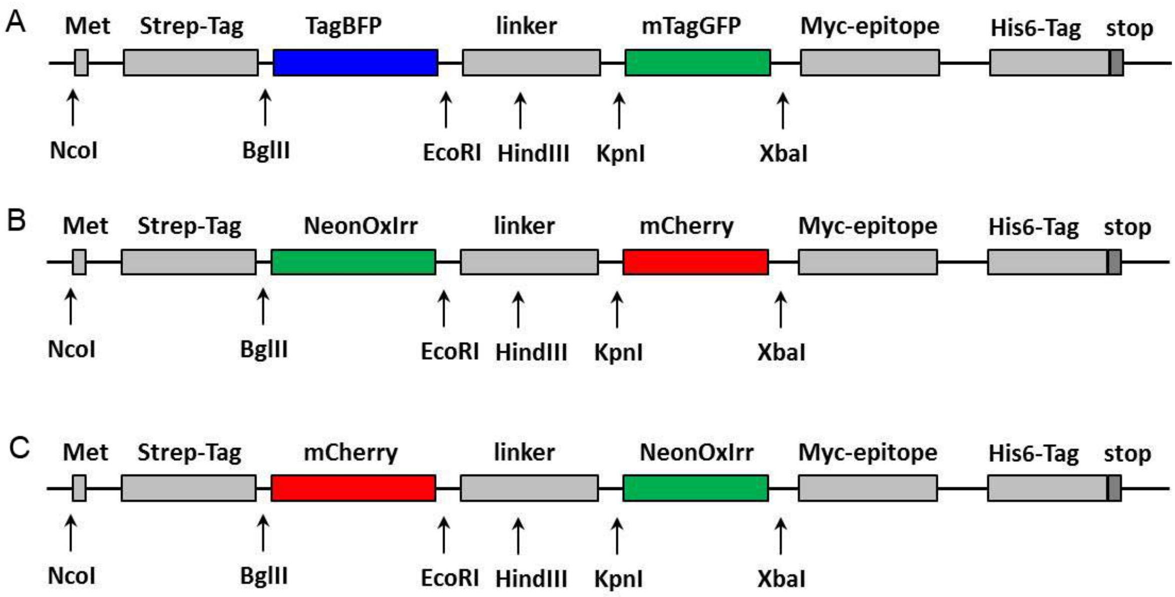

Figure S16. Schematic representation of the cloning sites in the original plasmid pBAD/Myc-HisB-mTagBFP-mTagGFP (A) and in the plasmids pBAD/Myc-HisB-NeonOxIrr-P2A-mCherry, pBAD/Myc-HisB-NeonOxIrr-L3- mCherry, pBAD/Myc-HisB-NeonOxIrr-L2-mCherry (B) and pBAD/Myc-HisB-mCherry-P2A-NeonOxIrr, pBAD/Myc-HisB-mCherry-L3-NeonOxIrr and pBAD/Myc-HisB-mCherry-L2 -NeonOxIrr (C).

Linker refers to the linkers P2A, L3 or L2 between fluorescent proteins NeonOxIrr and mCherry.

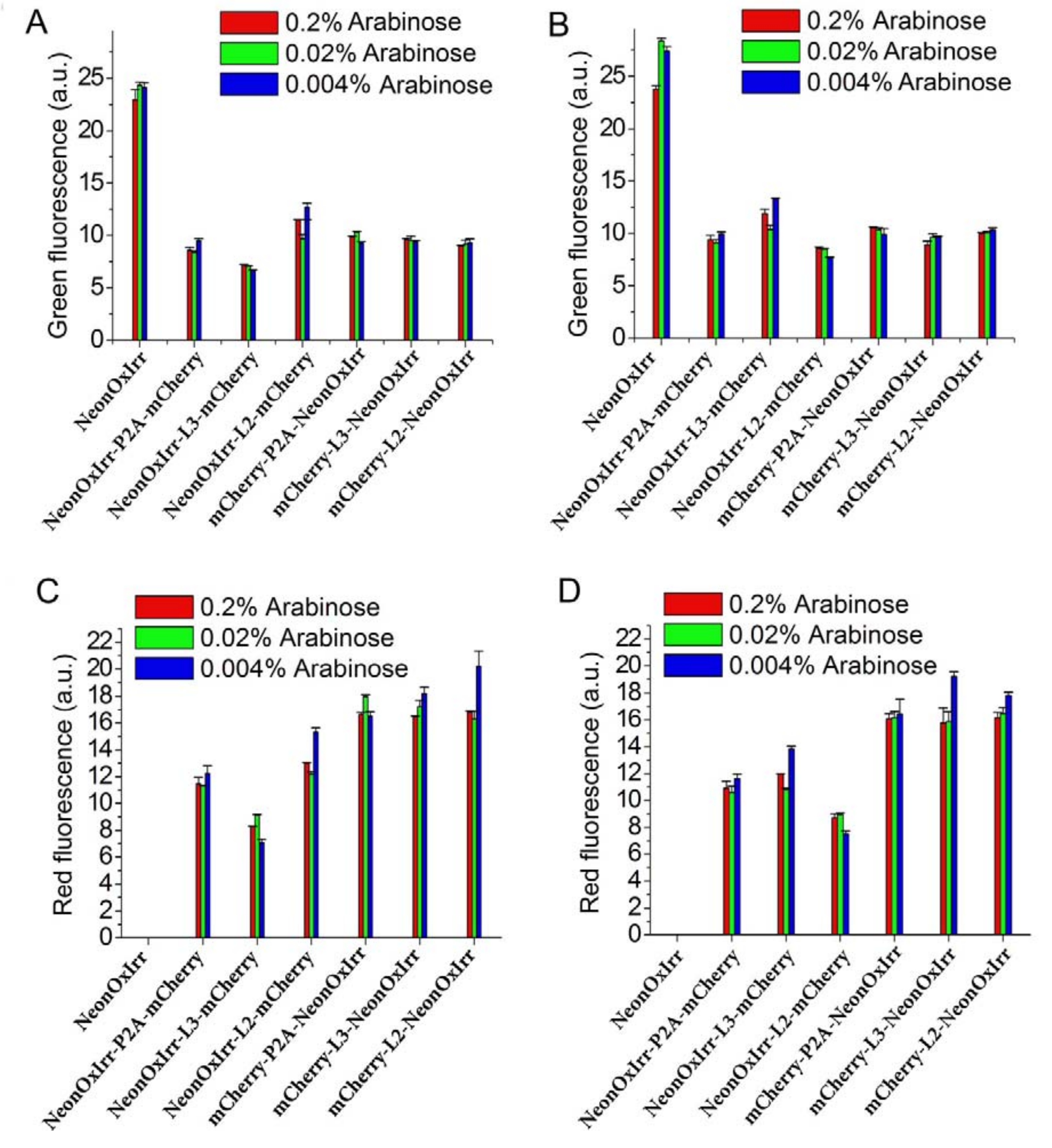

Figure S17. Optimizing maturation conditions in bacteria of NeonOxIrr and mCherry fusion proteins. Expression of fusion proteins was induced with the indicated concentrations of arabinose (A, C) for 24 hours at 37 degrees or (B, D) for 24 hours at 37 degrees, followed by incubation for 24 hours at 25 degrees.

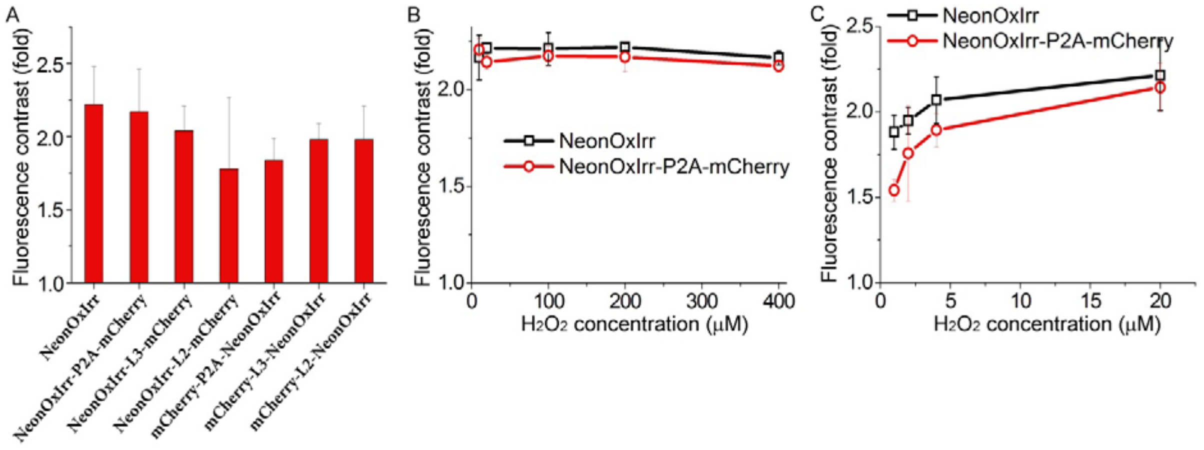

Figure S18. Fluorescence contrast of the NeonOxIrr sensor in fusion with the mCherry protein, depending on the order of the proteins in fusion, the length of the linkers between them (A) and the concentration of H_2O_2 in bacterial expression (B-C). The contrast was determined as the ratio of the maximum fluorescence after the addition of H_2O_2 to the fluorescence before addition of H_2O_2. Hydrogen peroxide was added to a final concentration of 200 μM (a), 10-400 μM (b) and 1-20 μM (c).

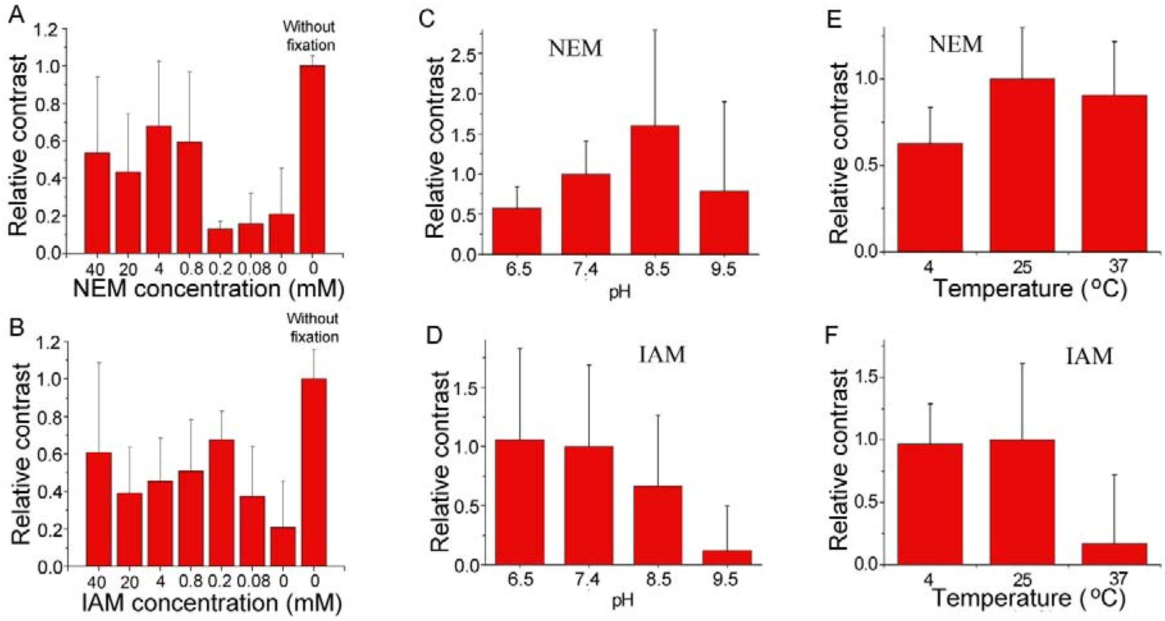

Figure S19. Dependence of the fluorescence contrast of the NeonOxIrr-P2A-mCherry fusion in *E. coli* bacteria after alkylation-fixation on the concentration of the NEM (A) and IAM (B)alkylating reagents; on the pH of the buffer for alkylation with 0.8 mM NEM (C) or 0.2 mM IAM (D); on the temperature of alkylation with 0.8 mM NEM (E) or 0.2 mM IAM (F). The contrasts correspond to oxidation of the sensor 200 μM H_2O_2 for 2 minutes and are normalized to contrast without fixation (A-B), fixed at pH 7.4 (C-D) and fixed at 25 degrees (E-F). Fixation of bacteria with 4% parapharmaldehyde with pre-alkylation in PBS buffer, pH 7.4 (A-B); pH6.5, 7.4, 8.5 and 9.5 (C-D); pH 8.5 (E) or 7.4 (F) was performed according to the protocol described in the paragraph Materials and Methods.

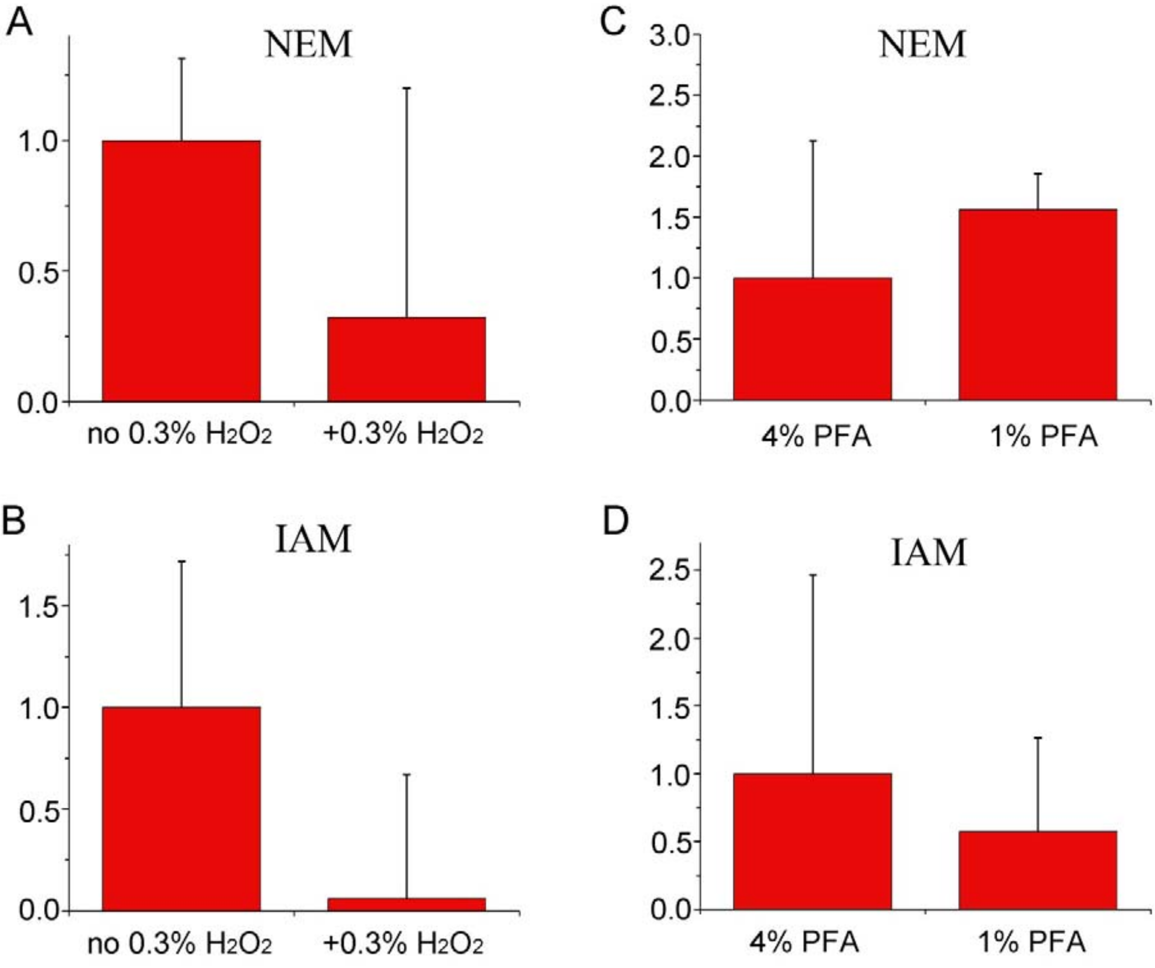

Figure S20. Change in the fluorescence contrast of the NeonOxIrr-P2A-mCherry sensor in *E.coli* bacteria after alkylation-fixation (A-B) with additional treatment with 0.3% hydrogen peroxide or (C-D) depending on the concentration of paraformaldehyde fixing agent (PFA). The contrasts correspond to the oxidation of the sensor 200 μM H2O2 for 2 minutes and are normalized to contrast without treatment with 0.3% hydrogen peroxide (A-B) or contrast when fixing with 1-4% PFA (C-D). Fixation of 4% and 1% PFA bacteria with pre-alkylation of 0.8 mM NEM in PBS buffer, pH 8.5 (A, C) or 0.2 mM IAM in PBS buffer, pH 7.4 (B, D) was performed according to the protocol described in Materials and Methods.

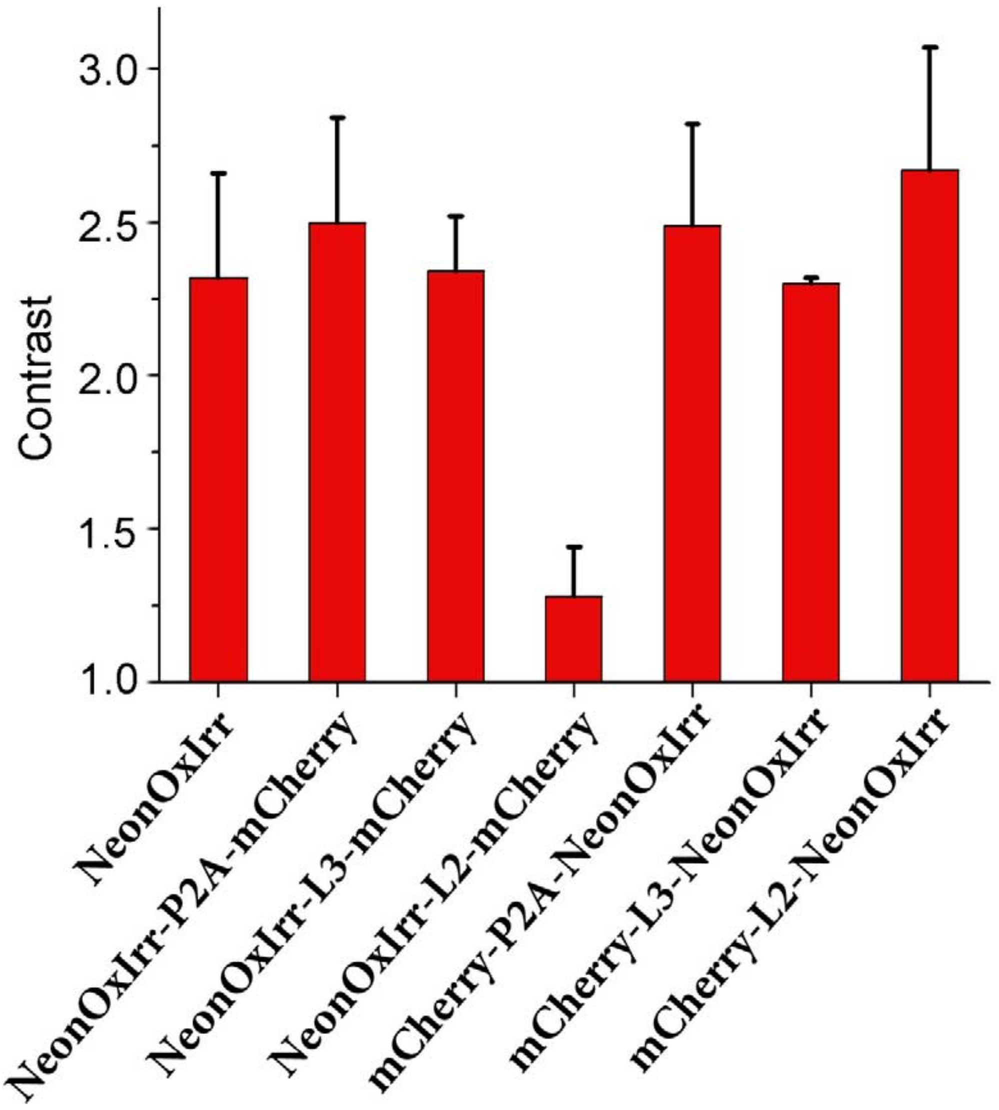

Figure S21. Dependence of fluorescence contrast of NeonOxIrr sensor in fusion with mCherry protein on the order of proteins in fusion and on the length of linkers between them when expressed in HeLa Kyoto mammalian cells. Green-red fusion sensors were expressed in the HeLa Kyoto mammalian cells. The fluorescent contrast was obtained by analyzing the confocal images of the cells before and after the addition of H_2O_2 in medium A to a 200 μM final concentration.

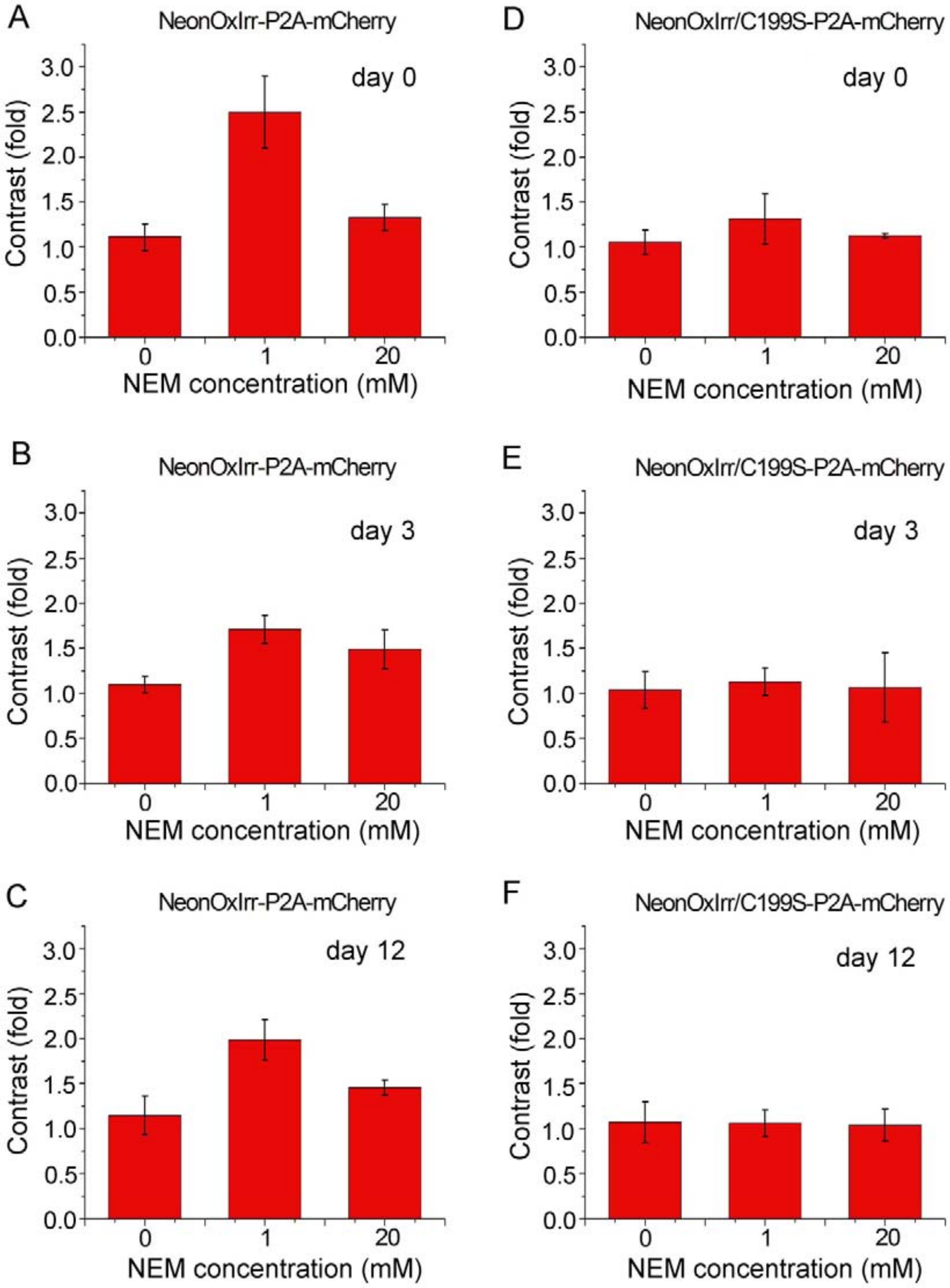

Figure S22. Dependence of the fluorescence contrast of the NeonOxIrr-P2A-mCherry sensor (A-C) and the NeonOxIrr mutant C99S-P2A-mCherry (D-F) in HeLa cells after alkylation with NEM and subsequent fixation with 4% PFA on the concentration of NEM through 0 (A, D), 3 (B, E), and 12 (C, F) days after fixation.

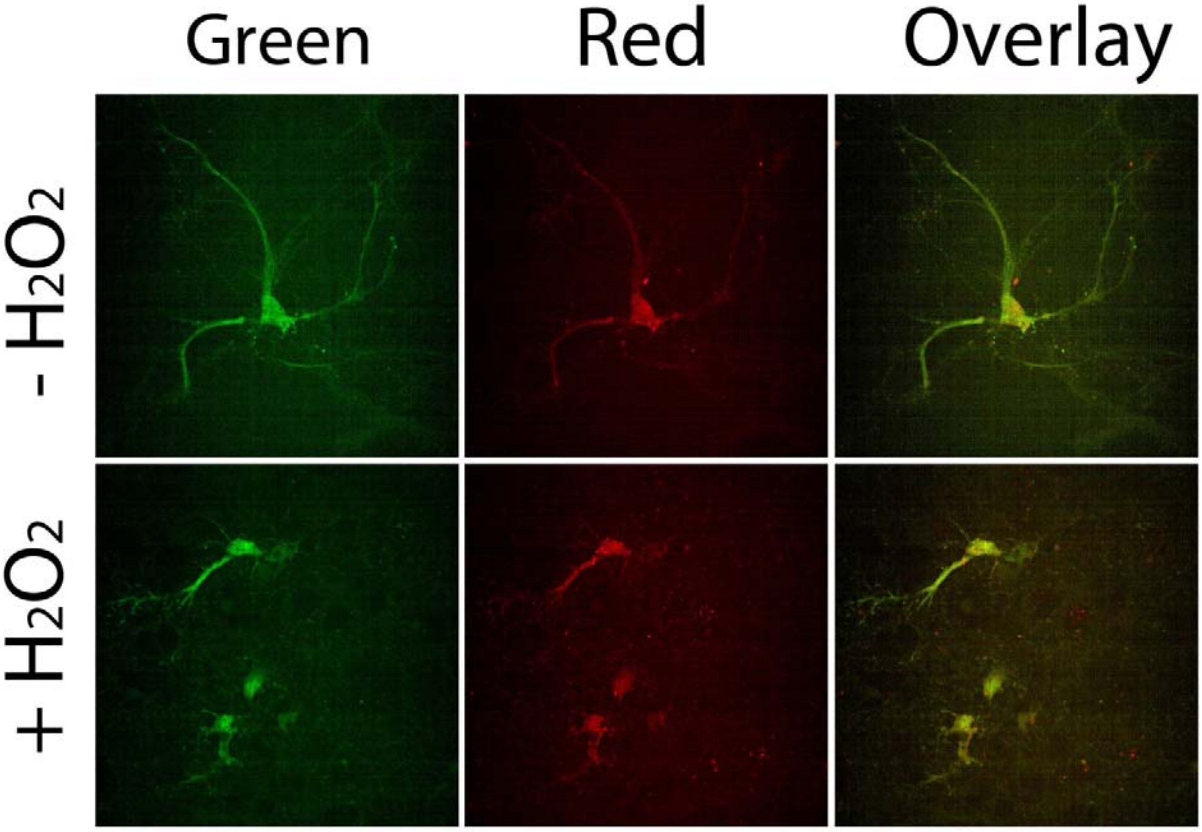

Figure S23. Ex vivo detection failure of the NeonOxIrr-P2A-mCherry sensor response to the addition of external hydrogen peroxide when expressed in neuronal dissociated culture cells after their NEM alkylation and fixation. An example of confocal images of cells of dissociated neuronal cultures after their alkylation and fixation with pre-incubation with 200 μM hydrogen peroxide for 5 minutes and without it.

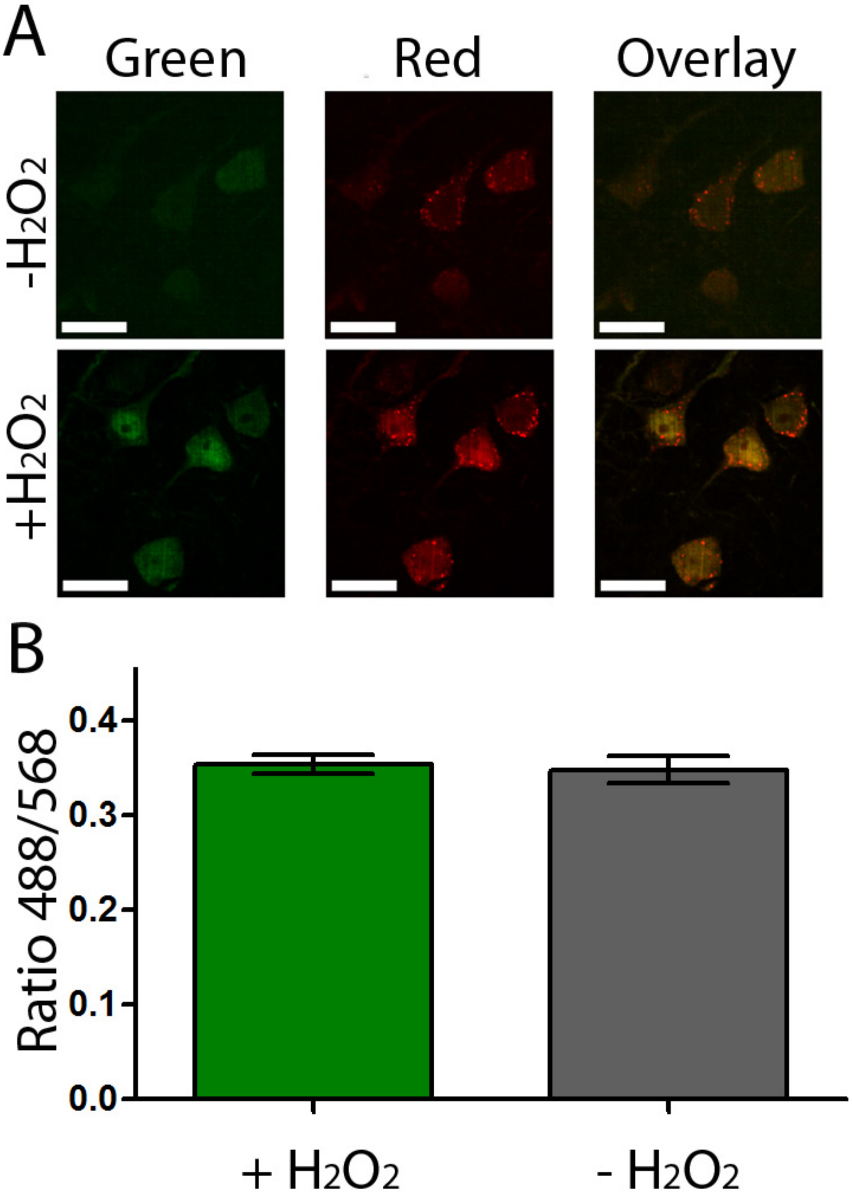

Figure S24. Ex vivo detection failure of the NeonOxIrr-P2A-mCherry sensor response to the addition of external peroxide to the somatosensory cortex of the mouse brain. (A) An example of confocal images of neuronal cells from slices of the somatosensory cortex of mouse brain. Slices were obtained after the extraction of brains from perfused animals. Perfusion was performed with 4% PFA pre-alkylated with 1 mM NEM for 10 minutes. Immediately prior to perfusion, 200 μM H_2O_2 (designated as + H_2O_2) was injected into only one of the two regions infected with rAAV particles encoding NeonOxIrr-P2A-mCherry; the infected area of the brain without the introduction of 200 μM H_2O_2 is designated as -H_2O_2. (B) Averaged green fluorescence normalized to red fluorescence (488/568 ratio) is shown for neuronal cells from areas of the somatosensory cortex of mice with an exposure to 200 μM H_2O_2 and without it. Scale bars are 20 μm.

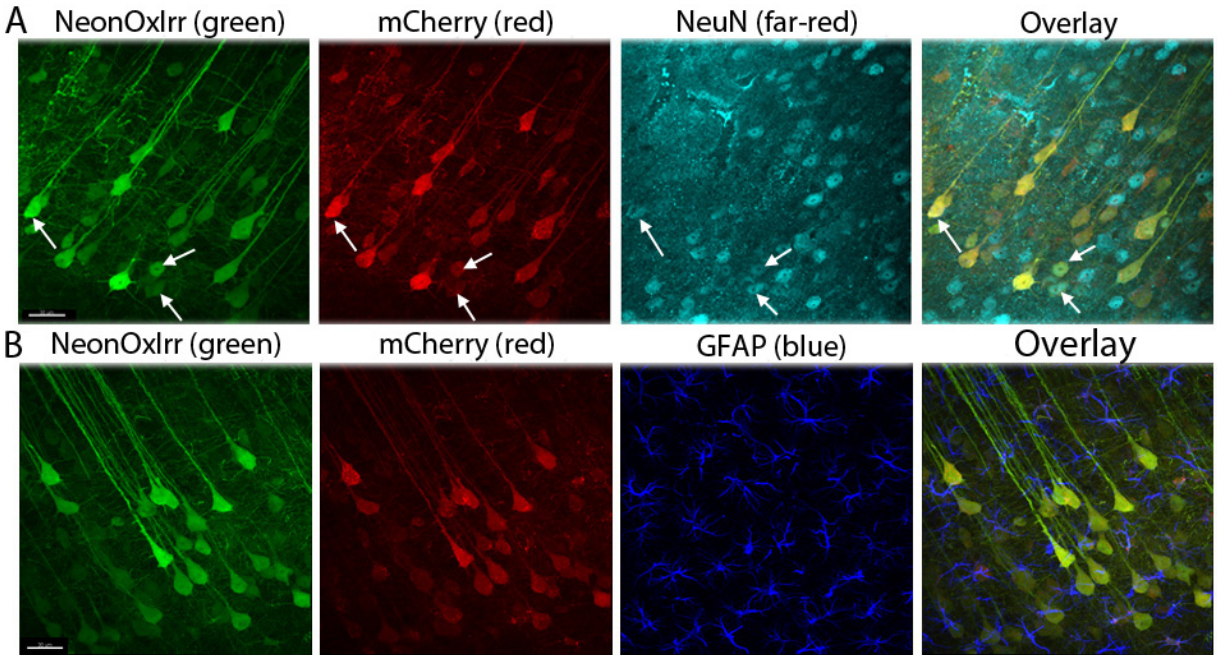

Figure S25. Visualization of mouse brain cortex slices after infection with adeno-associated particles encoding the fusion protein gene NeonOxIrr-P2A-mCherry, followed by alkylation with 1 mM NEM, fixation with 4% paraformaldehyde by perfusion, preparation of fixed-brain slices and their immunohistochemical staining with neuron NeuN (A) and astrocyte GFAP (B) markers. (A) Images of green NeonOxIrr (excitation at 488 nm), red mCherry (excitation at 561 nm), far-red NeuN (excitation at 647 nm), shown by cyan color, and the overlay of fluorescent images (see also ESI Video S3). For example, white arrows indicate neurons in which NeuN is colocalized with NeonOxIrr-P2A-mCherry. (B) Images of green NeonOxIrr (excitation at 488 nm), red mCherry (excitation at 561 nm), blue GFAP (excitation at 405 nm), and the overlay of fluorescent images (see also ESI Video S4). Scale bars – 30 μm.

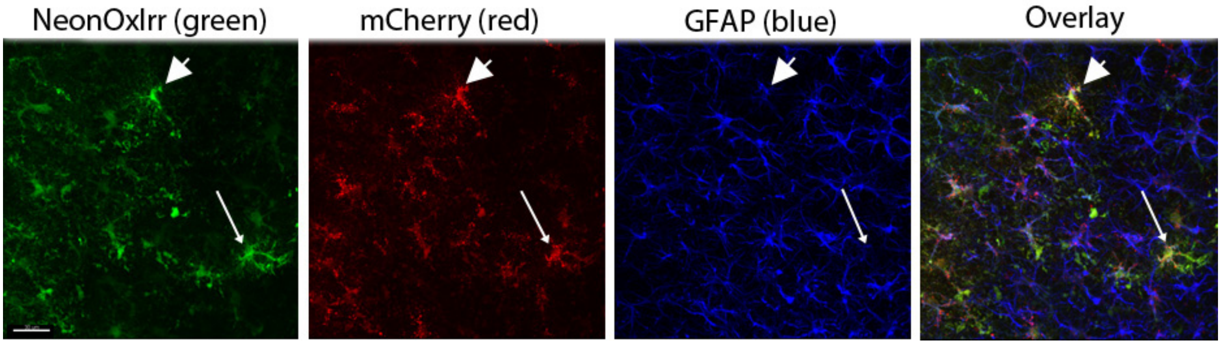

Figure S26. Visualization of cortex slices of mouse brain obtained after infection with adeno-associated particles encoding the fusion protein gene NeonOxIrr-P2A-mCherry, followed by alkylation with 1 mM NEM, fixation with 4% paraformaldehyde by perfusion, preparation of fixed brain slices and their immunohistochemical staining with the marker of astrocytes GFAP. Images of green NeonOxIrr (excitation at 488 nm), red mCherry (excitation at 561 nm), blue GFAP (excitation at 405 nm), and the overlay of fluorescent images. The arrow and the indicator mark cells expressing NeonOxIrr-P2A-mCherry. A white thin arrow indicates an amoeboid-like GFAP-negative cell, which is the cell of activated microglia; the indicator indicates an activated astrocyte having large soma and high GFAP expression. Scale bar – 30 μm.

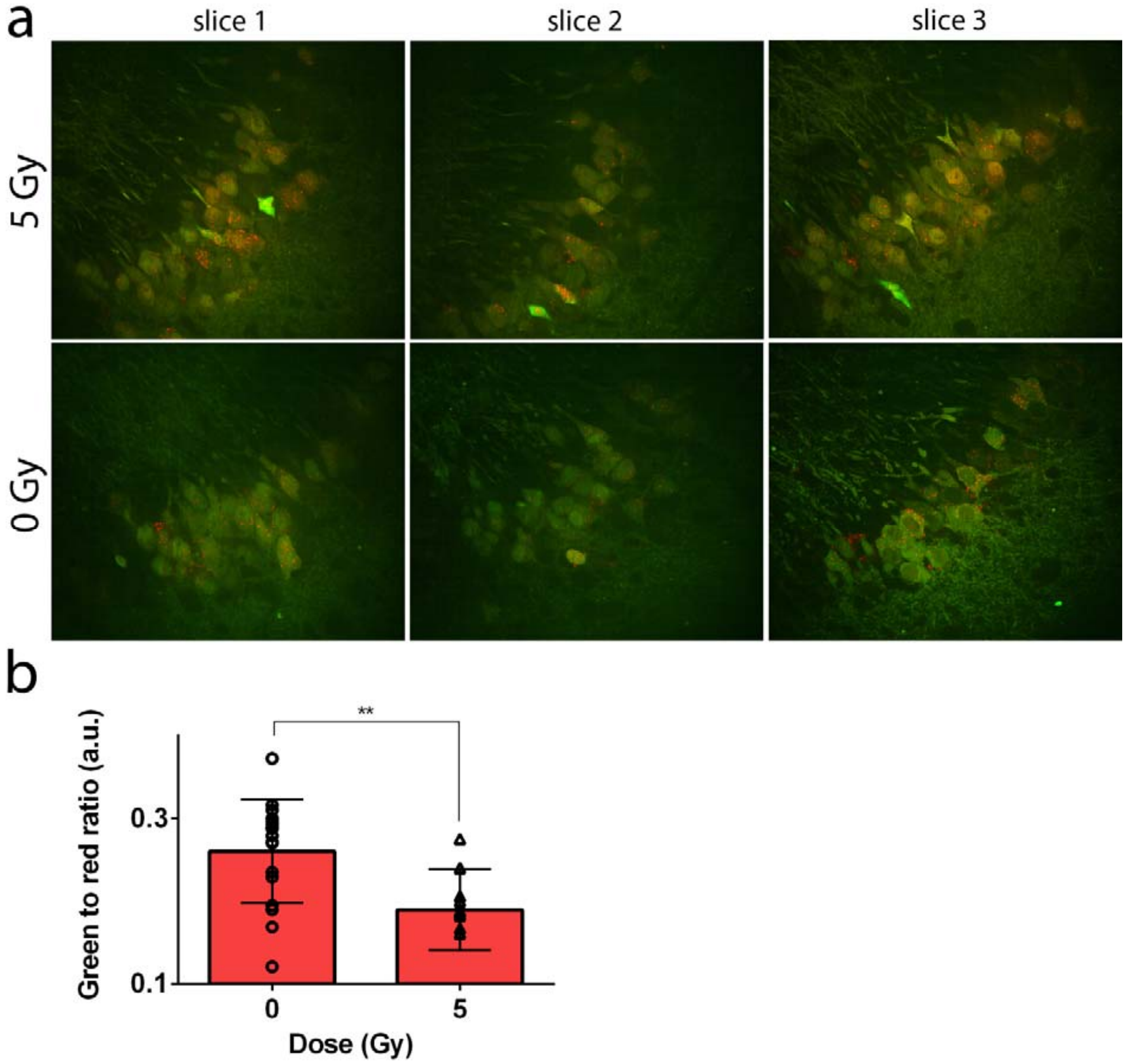

Figure S27. ***Ex vivo*** **detection of the NeonOxIrr-P2A-mCherry indicator response to 5 Gy gamma irradiation in the cortex of mice.** (A) Confocal images of cortical cortex transduced with rAAV-CAG-NeonOxIrr-P2A-mCherry virus particles are shown for non-irradiated control mice and 5 Gy gamma-irradiated mice. (B) The mean and maximum values of the normalized ratios of green fluorescence to red fluorescence (488/561) are shown for neuronal cells from confocal images of cortical areas without radiation and 5 Gy gamma irradiation. Slices were obtained after removing the brains from the perfused animals. 35 minutes after irradiation perfusion was performed with 4% PFA with pre-alkylation of 1 mM NEM for 10 minutes.

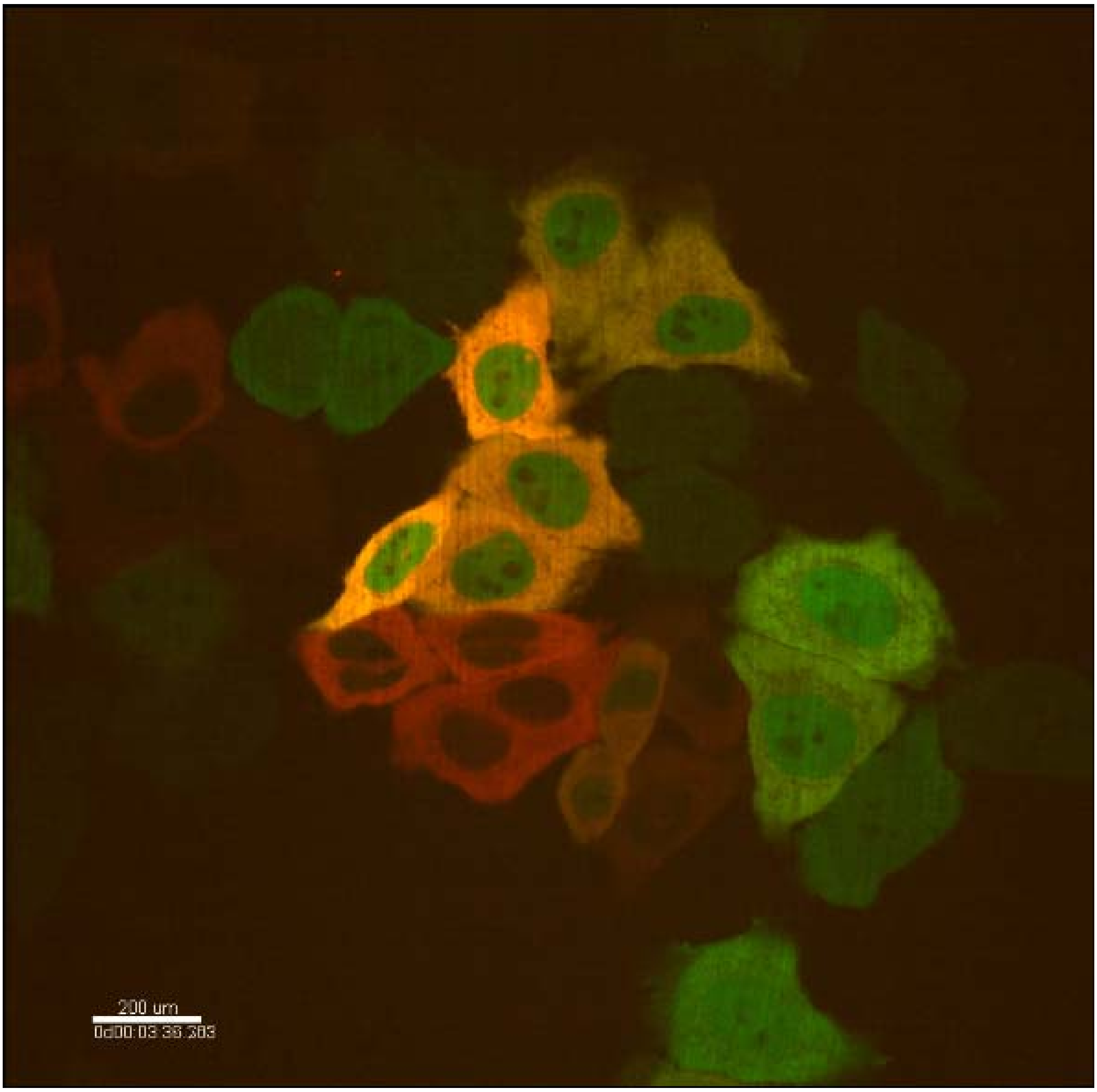

Video S1. Simultaneous two-color imaging of H_2O_2 and calcium transients in HeLa cells. Expanded data to Figure 2d in the Main text. HeLa Kyoto cells were transiently co-transfected with pLU-CMV-NeonOxIrr and pAAV-CAG-NES-R-GECO1 plasmids and H_2O_2 (200 μM) and ionomycin (2.5 μM) were consequently added in 1 and 3 min after the beginning of the confocal imaging, respectively. The frame at 3.5 min is shown.

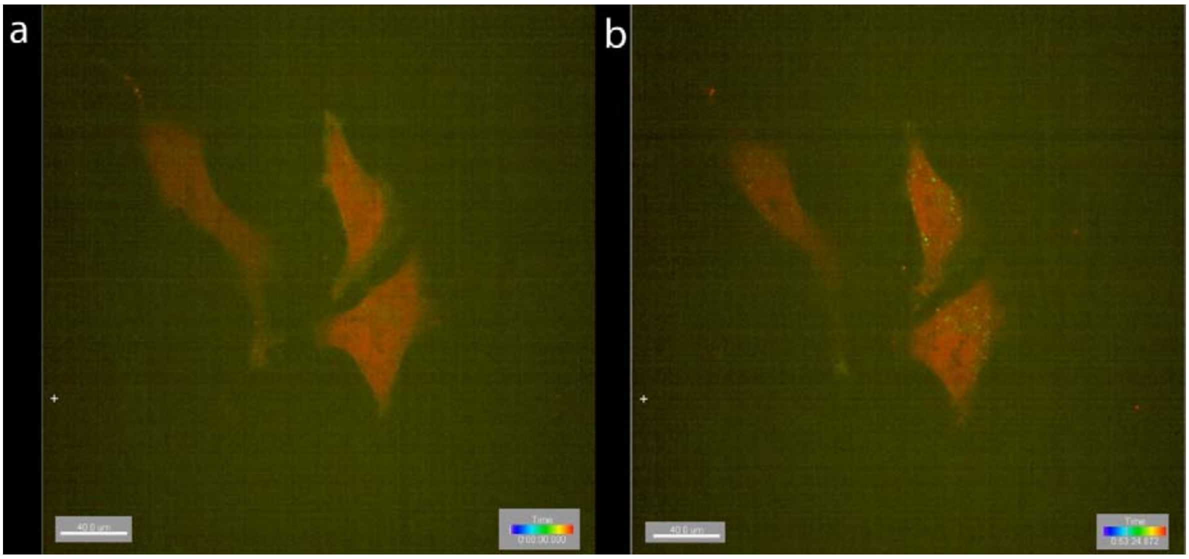

Video S2. EGF-induced endocytosis of EGFR-GFP fusion in HeLa cells. HeLa Kyoto cells were transiently co-transfected with pN1-CMV-EGFR-GFP and pLU-CMV-R-GECO1 plasmids and EGF (100 ng/ml) was added in 3 min after the beginning of the confocal imaging. The frames at 0 and 53 min are shown.

Table S1. **Fluorescence contrasts and reduction half-times for redox sensors in *E.coli* bacterial suspensions.**

Protein	Concentration of H_2O_2, μM											
	1		2		10		20		100		200	
	Cont., fold	*$t_{½red}$, min*	*Cont., fold*	*$t_{½red}$, min*	*Cont., fold*	*$t_{½red}$, min*	*Cont., fold*	*$t_{½red}$, min*	*Cont., fold*	*$t_{½red}$, min*	*Cont., fold*	*$t_{½red}$, min*
**NeonOxIrr**	1.3	ND	1.4	ND	2.6	290	2.8	277	2.7	275	2.7	280
**Hyper3**	1.16	ND	1.15	ND	1.9	52	2.0	55	2.5	62	2.5	61

ND, not determined

Table S2. Oxidation rates (k_{+1}, $M^{-1}sec^{-1}$) for purified H_2O_2 sensors.

Oxidant / Protein	H_2O_2 [1]	H_2O_2 [1] 37°C	O_2 [2]
**NeonOxIrr**	16000±1000	22100±300	3.4
**HyPer3**	780±30	1443±100	0.19

Purified proteins were reduced with 70mM DTT. [1] k_{+1} oxidation rates were determined in the presence of varying concentrations of H_2O_2 of 0.1-16000 μM. K_{+1}' values (in sec^{-1}) at each H_2O_2 concentration were calculated as initial slope on the linear part of $\ln[(F_{max}-F_{min})/(F_{max}-F(t))]$ vs time dependence, where F_{max}, F_{min} and F(t) are maximal, minimal and at given time fluorescence intensities values, respectively. k_{+1} oxidation rates (in $M^{-1}sec^{-1}$) were calculated as a slope of K_{+1}' vs H_2O_2 concentration dependence. [2] Oxidation rates were determined in the absence of H_2O_2 and in the presence of 0.005mg/ml of catalase. Rates were estimated suggesting concentration of atmospheric oxygen of 250 μM.

Table S3. **Effective oxidation rates (k_{+1}, $M^{-1}sec^{-1}$) and reduction half-times for redox indicators in cytoplasm of live HEK293T cells upon addition of the external H_2O_2.**

Protein	k_{+1}, $M^{-1}sec^{-1}$ [a]	$t_{1/2\ red}$, min [b]
**NeonOxIrr**	137±15	44±16
**HyPer3**	79±6	5.8±1.4

[a] Oxidation rates were determined in DMEM/10%FBS/Glutamine in the presence of varying final concentrations of external H_2O_2 of 5-200 μM. K_{+1}' values (in sec^{-1}) at each H_2O_2 concentration were calculated as initial slope on the linear part of $\ln[(F_{max}-F_{min})/(F_{max}-F(t))]$ vs time dependence, where F_{max}, F_{min} and F(t) are maximal, minimal and at given time fluorescence intensities values, respectively. k_{+1} oxidation rates (in $M^{-1}sec^{-1}$) were calculated as a slope of K_{+1}' vs H_2O_2 concentration dependence. [b] Reduction half-times observed after addition of external H_2O_2 till final concentration of 200 μM are shown. After addition of H_2O_2 when sensors were maximally oxidized and reached their maximal fluorescence we added catalase (5 μg/ml final concentration) for H_2O_2 degradation.

Table S4. Sensitivity of redox sensors expressed in the cytoplasm of HEK293T cells to the external H_2O_2.

H_2O_2 concentration of, μM	Fluorescence contrast (fold)	
	**NeonOxIrr**	**HyPer3**
2	ND	ND
5	111±4	112±5
20	127±4	125±11
50	140±16	150±24
200	232±34	280±23

ND, not detectable. Fluorescence contrasts observed at addition of external H_2O_2 till final concentration of 2-200 μM are shown.

Table S5. Fluorescence contrasts of redox sensors expressed in different organelles of HEK293T cells to external H_2O_2.

Protein	Cytoplasm	Nucleus	Peroxisomes	Mitochondria		Membrane	Golgi	ER
				Lumen	IMS			
**NeonOxIrr**	232±34	245±51	149±53	244±67	227±23	128±10	117±11	121±8
**HyPer3**	279±24	ND						

ND, not determined

Table S6. List of primers.

**mCherry-KpnI**	5′- TCTGGTACCATGGTGAGCAAGGGCGAG
**mCherry-XbaI-r**	5′- TGTTCTAGAGCCTTATACAGCTCGTCCATGC
**P2A-EcoRI**	5′- TTGAATTCCGACCGGTTCACGTGGGAAG
**P2A-HindIII-r**	5′- CCAAGCTTACTAGTCACGTGTGGTCC
**Fw-BglII-TagBFP**	5′- GC TCG AGA TCT ATG AGC GAG CTG ATT AAG GAG
**L2**	5′- gcacaagcTTGAATTCGGAGGCAGTGGATCCaagcttggtggttctggttc
**L2-r**	5′- GAACCAGAACCACCAAGCTTGGATCCACTGCCTCCGAATTCAAGCTTGTGC
**L3**	5′- CTTGAATTCGGTGGTTCTGGTTCTGGAGGGAGTGGCAGCAAGCTTGGTGGTTC TGGTTCTGGTACCATG
**L3-r**	5′- CATGGTACCAGAACCAGAACCACCAAGCTTGCTGCCACTCCCTCCAGAACCA GAACCACCGAATTCAAG
**Fw-BglII-Hyper**	5′-GCTCGAGATCTATGGAGATG GCAAGCCAGCAG
**Ox-EcoRI-r**	5′-ccgaattcaaccgcctgttttaaaac
**Fw-LSSmOrange-BglII**	5′-gacAGATCTATGGTGAGCAAGGGCGAGGAG
**mCherry-EcoRI-r2**	5′-ccgaattcCTTATACAGCTCGTCCATGC
**L-KpnI-Neon**	5′- GAGGGGGTTCCGGCGGTGGAGGTAGCGGTGGTGGAGGCTCGGGCGGAGGTA CCATGGAGATGGCAAGCCAG
**Ox-XbaI-r**	5′-TGTTCTAGAGCaaccgcctgttttaaaac
**NeonOx-AscI**	GAC GGC GCG CCA CCA TGG CTA GCC TCG AGA TGG AGA TGG CAA GCC AGC AG
**mCherry-BsrGI-r**	ACTTGTACATTACTTATACAGCTCGTCCATGC
**mCher-Bam-Asc-Sal-Nhe**	ccaGGATCCGGCGCGCCgtcgacaccATGGCTAGCCTCGAGATGGTGAGCAAGGG CGAG
**NeonOx-BsrGI-r**	ACT TGT ACA TCT AGA CGG ATA CCG GTT TAA ACC GCC TGT TTT AAA ACT TTA TC

Scheme S1. **The recommended alkylation-fixation protocols for the possible applications.**

Application	Recommended protocol	
	**Alkylation**	**Fixation**
**Bacteria**	10 min, 1 mM NEM, PBS (pH 8.5)	20 min, 1-4% PFA, PBS
**HeLa cells**	10 min, 1 mM NEM, DPBS	15-20 min, 4% PFA, PBS
**Neuronal cultures**	10 min, 0.01% NP-40, 1 mM NEM, PBS	15-20 min, 4% PFA, PBS
**Mouse brain**	10 min perfusion with 70 ml of 0.01% NP-40, 1 mM NEM, PBS	perfusion with 30 ml of 4% PFA, PBS; brains post-fixation for overnight, at 4°C, 4% PFA, PBS

Supplementary Materials and Methods

Mutagenesis and screening of libraries

For the construction of original library with randomized linkers between the cpmNeonGreen and OxyR parts we used an overlap-extension approach[4]. An library of genes coding redox sensors mutants was amplified as a *Bgl*II-*Eco*RI fragment, using a polymerase chain reaction, and inserted into a pBAD/His-B vector (Invitrogen). A site-specific mutagenesis of the genes was performed using a QuickChange Mutagensis Kit (Stratagene) or an overlap-extension approach[4]. A random mutagenesis was performed using a GeneMorph II Random Mutagenesis Kit (Stratagene) or a Diversity PCR Random Mutagenesis Kit (Clontech) under conditions resulting in a mutation frequency of up to 16 mutations per 1,000 base pairs. After mutagenesis, a mixture of mutated genes was electroporated into BW25113 bacterial host cells (CGSC Collection).

Libraries of 10^6-10^7 independent clones of the redox sensors mutants were oxidized with 200 μM final concentration of H_2O_2 and screened using a FACS Aria3 (BD Biosciences, USA) fluorescence-activated cell sorter (FACS), followed by colony visualization and screening, using a Leica M205FA (Leica, Germany) fluorescence stereomicroscope equipped with DFC310FX camera (Leica Microsystems, Germany) and mercury metal halide light source EL6000 (Leica Microsystems, Germany) (as described below). After each round of FACS screening, typically 10-20 best reacting candidate clones were sequenced, purified, and characterized before the next round of mutagenesis.

Alternatively, each round of screening on Petri dishes included generation of bacterial library of mutants containing 2-3 random mutations per gene of 1.5 kbps length using random mutagenesis. Screening of the $2*10^4$ mutants from library on Petri dishes was further performed under a Leica M205FA (Leica, Germany) fluorescence stereomicroscope by means of selection of the brightest and the most contrast clones reacting on spraying of 10 mM H_2O_2. Green fluorescence was registered by 480/40BP excitation (75 $\mu W/cm^2$ on the sample) and 540/40BP emission filters, respectively. Images obtained were analyzed using ImageJ software. We next performed analysis of brightness and contrast of several tens of best selected mutants on bacterial suspension with 96-well Modulus™ II Microplate Reader (Turner Biosystems, USA). Characterization of brightness of several best variants in oxidized state was further performed on pure protein. The best mutant(s) were utilized as template(s) for the next round(s) of molecular evolution. Final versions of sensors were purified on Ni-NTA agarose for His_6-tag as described [5].

Cloning of the fusion proteins of NeonOxIrr with mCherry in the bacterial system

The mCherry red fluorescent protein gene was amplified by PCR from the plasmid pBAD/HisB-mCherry (courtesy of V.Verhusha, Albert Einstein College, USA) as the KpnI-XbaI fragment (ESI Table S1). Next, the KpnI-mCherry-XbaI restriction product was inserted by ligation with T4 DNA ligase into the pBAD/Myc-HisB-mTagBFP-mTagGFP vector [6] (courtesy of V.Verkhusha, Albert Einstein College, USA) in place of the mTagGFP gene (ESI Figure S13) to obtain a final plasmid called pBAD/Myc-HisB-mTagBFP-mCherry. Further, the linker between mTagBFP-mCherry proteins from the source vector pBAD/Myc-HisB-mTagBFP-mTagGFP (ESI Figure S13), having EcoRI, HindIII and KpnI restriction sites, was replaced by three other linkers, namely P2A (EFRPVHVGSGATNFSLLKQAGDVEENPGPHVTSKLGGSGSGT), L3 (EFGGSGSGGSGSKLGGSGSGT) and L2 (EFGGSGSKLGGSGSGT) having a length along with restriction sites EcoRI/KpnI 42, 21 and 16 amino acids, respectively. To obtain the pBAD / Myc-HisB-mTagBFP-P2A-mCherry vector, the self-cleavable P2A peptide derived from the porcine teschovirus-1 virus [7] was amplified from plasmid

pAAV-CAG-PAiRFP1-P2A-EGFP (kindly provided from Edward Boyden's laboratory, MIT, USA) as the EcoRI- HindIII fragment and inserted at the EcoRI/HindIII restriction sites into the plasmid pBAD/Myc-HisB-mTagBFP-mCherry. The L2 linker was inserted into the plasmid pBAD/Myc-HisB-mTagBFP-mCherry by PCR with overlapping fragments [4] (ESI Table S1), to form the plasmid pBAD/Myc-HisB-mTagBFP-L2-mCherry. The L3 linker was inserted into the pBAD/Myc-HisB-mTagBFP-mCherry plasmid by overlapping PCR (ESI Table S1) to form plasmid pBAD/Myc-HisB-mTagBFP-L3-mCherry.

To obtain plasmids pBAD/Myc-HisB-NeonOxIrr-P2A-mCherry, pBAD/Myc-HisB-NeonOxIrr-L2-mCherry and pBAD/Myc-HisB-NeonOxIrr-L3-mCherry (ESI Figure S13), the NeonOxIrr gene was amplified by PCR from the plasmid pBAD/HisB-NeonOxIrr (ESI Table S1). The BglII-NeonOxIrr-EcoRI restriction product was then inserted by ligation with T4 DNA ligase into the corresponding vector pBAD/Myc-HisB-mTagBFP-X-mCherry (where X is the P2A, L2 or L3 linker) at the BglII/EcoRI restriction sites in place of the mTagBFP gene .

To obtain plasmid pBAD/Myc-HisB-mCherry-X-mCherry (where X is the linker P2A, L2 or L3), the mCherry red fluorescent protein gene was amplified from pBAD/HisB-mCherry using PCR as the BglII- EcoRI fragment (ESI Table S1). The BglII-mCherry-EcoRI restriction product was then inserted by ligation with T4 DNA ligase into the corresponding pBAD/Myc-HisB-mTagBFP-X-mCherry vector (where X is P2A, L2 or L3) at the BglII/EcoRI restriction sites in place of the mTagBFP gene.

To obtain plasmids pBAD/Myc-HisB-mCherry-P2A-NeonOxIrr, pBAD/Myc-HisB-mCherry-L2-NeonOxIrr and pBAD/Myc-HisB-mCherry-L3-NeonOxIrr (ESI Figure S13), the NeonOxIrr gene was amplified by PCR as the KpnI-XbaI fragment (ESI Table S1). The KpnI-NeonOxIrr-XbaI restriction product was then inserted by ligation with T4 DNA ligase into the corresponding pBAD/Myc-HisB-mCherry-X-mCherry vector (where X is the P2A, L2 or L3 linker) at the KpnI/XbaI restriction sites instead of the second copies of the mCherry gene.

Expression of fusion proteins in bacterial culture

Seven plasmids pBAD/HisB-NeonOxIrr, pBAD/Myc-HisB-NeonOxIrr-P2A-mCherry, pBAD/Myc-HisB-NeonOxIrr-L3-mCherry, pBAD/Myc-HisB-NeonOxIrr-L2-mCherry, pBAD/Myc-HisB-mCherry-P2A-NeonOxIrr, pBAD/Myc-HisB-mCherry-L3-NeonOxIrr and pBAD/Myc-HisB-mCherry-L2-NeonOxIrr, encoding fusion proteins with six histidine residues at the N-terminus, were transformed into bacteria BW25113 (CGSC Collection), not consuming arabinose. Further, the bacteria were grown in LB medium at different concentration of arabinose inducer 0.2, 0.02 and 0.004% and temperature 37°C for 24 h or 37°C for 24 h, followed by incubation at 25°C for 24 h. Red and green fluorescence were measured in bacterial suspensions in 100 mM NaOAc, pH 7.4 buffer to neutralize the effect of pH.

Protein purification and characterization

The bacterial cultures carrying pBAD/HisB-NeonOxIrr plasmid were grown in LB medium supplemented with 0.002% arabinose and 100 μg/ml ampicillin overnight at 37°C and 220 rpm. The cultures were then centrifuged at 4648 g for 10 min, and the cell pellets were re-suspended in PBS at pH 7.4 with 300 mM NaCl and lysed by sonication on ice. The recombinant proteins were purified using Ni-NTA resin (Qiagen, USA), followed by dialysis for 12-16 h against PBS, pH 7.4. The absorbance values and excitation and emission spectra were measured with a CM2203 spectrofluorometer (Solar, Belarus).

Chromophore extinction coefficient for purified NeonOxIrr was measured in PBS, pH7.4 by alkaline denaturation with 1 M NaOH and using extinction coefficient for GFP-like chromophore equal to 44,000 M^{-1} cm^{-1} in 1 M NaOH [8].

For quantum yield determination, the integrated fluorescence values of purified NeonOxIrr were measured in PBS, pH7.4, as previously reported [5]. mEGFP was used as a reference standard.

For determination of the pH dependence, NeonOxIrr in the oxidized state (PBS, pH7.4) or in the reduced state (PBS, pH7.4 supplemented with 14 mM DTT) were diluted 1:100 into a series of pH adjusted buffers (100 mM NaOAc, 300 mM NaCl for pH 3-5.0, and 100 mM NaH_2PO_4, 300 mM NaCl for pH 4.5-9.0) with pH values ranging from 9 to 3 in 0.5 pH units interval in a 96-well black clear bottom plate (Thermo Scientific, USA). Fluorescence was measured using a Modulus™ II Microplate Reader (TurnerBiosystems, USA).

Photobleaching experiments were performed with suspensions of purified proteins in mineral oil, as previously described [5]. Briefly, the kinetics of photobleaching was measured using purified proteins dialyzed in PBS, pH 7.4, in aqueous microdroplets in mineral oil using Zeiss Axio Imager Z2 microscope (Zeiss, Germany) equipped with a 120 W mercury short-arc lamp (LEJ, Germany), a 63× 1.4 NA oil immersion objective lens (PlanApo, Zeiss, Germany), a 470/40BP excitation filter, a FT 495 beam splitter, and 525/50BP emission filters. Light power density (7.3 mW/cm^2) was measured at a rear focal plane of the objective lens using PM100D power meter (ThorLabs, Germany) equipped with S120VS sensor (ThorLabs, Germany). The times to photobleach from 1000 down to 500 emitted photons per second were calculated according to standard procedures [9]. In brief, the averaged raw data were corrected for a spectral output of the lamp, transmission profiles of the excitation filter and dichroic mirror, and absorbance spectra of the respective green fluorescent proteins and their quantum yields. mEGFP protein was used as a reference.

To study protein maturation, BW25113 bacteria transformed with the pBAD/HisB-NeonOxIrr, pBAD/HisB-Hyper3 or pBAD/HisB-mEGFP plasmids were grown in 150 ml of LB medium supplemented with ampicillin at 37°C overnight. The next morning 0.2% arabinose was added to bacterial cells. Upon induction of protein expression, bacterial cultures were grown at 37°C in 50 ml tubes filled to the brim and tightly sealed to restrict oxygen supply. After 2 hours, the cultures were centrifuged in the same tightly closed tubes. After opening the tubes, the bacteria were sonicated in PBS buffer and the resulting proteins were purified using Ni-NTA resin within 10 min, with all procedures and buffers at or below 4°C. Protein maturation occurred in PBS at 37°C. Green fluorescence signal of the proteins was monitored using a CM2203 spectrofluorometer (Solar, Belarus).

Immunohistochemical staining

Staining was performed without prior unmasking. The fixed slices were washed with PBS. Then, permeabilization and blocking of non-specific antibody binding were performed by incubating the slices in PBS containing 2% Triton X-100 and 5% normal goat serum at room temperature for 1 hour. The slices were then washed three times with PBS containing 0.2% Triton X-100. Incubation with primary antibodies by murine anti-NeuN (1:100, Millipore, MAB377) and rabbit anti-GFAP (1:500, Life Technology, 180063) was performed at room temperature for 12 hours. A solution of primary antibodies was prepared using PBS containing 0.2% Triton X-100, 5% normal goat serum. The slices were then washed three times with PBS containing 0.2% Triton X-100. Incubation with secondary antibodies goat anti-rabbit Alexa 647 (1:500, Life Technologies, A-21245), goat anti-rabbit DyLight 405 (1:250, Life Technologies, 35550) and goat anti-mouse Alexa 647 (1:500, Life Technologies, A-21236) were conducted in the dark at room temperature for 2 hours. A solution of secondary antibodies was prepared using PBS containing 0.2% TritonX-100, 5% normal goat serum. The slices were then washed three times with PBS containing 0.2% TritonX-100 and three times with PBS. After completion of all staining, the brain slices were glued onto a slide and encased under coverslips using a special medium for fluorescent preparations (Dako, Dako). The enclosed slices were stored at 4°C in the dark. The images were obtained

on a scanning confocal microscope Leica SP5 by scanning along the Z axis in steps of 1 μm and a resolution of 1024 × 1024. To excite fluorescent dyes in the preparations, 4 laser lines were used: 405, 488, 561 and 640 nm. Scanning was carried out with the use of 20×, NA0.7 and oil-immersive 63×, NA1.4 lenses. Acquiring of three-dimensional images and analysis of colocalization was carried out in the program module Imaris (Bitplane, Switzerland).

Supplementary Results

Selection of the optimal linker, conditions for bacterial expression and oxidation by hydrogen peroxide

To test the expression conditions of the ratiometric versions of the NeonOxIrr sensor in bacteria, we varied the concentration of the inductor-arabinose and the temperature. 7 plasmids pBAD/HisB-NeonOxIrr, pBAD/Myc-HisB-NeonOxIrr-P2A-mCherry, pBAD/Myc-HisB-NeonOxIrr-L3-mCherry, pBAD/Myc-HisB-NeonOxIrr-L2-mCherry, pBAD/Myc-HisB-mCherry-P2A-NeonOxIrr, pBAD/Myc-HisB-mCherry-L3-NeonOxIrr and pBAD/Myc-HisB-mCherry-L2-NeonOxIrr were transformed into BW25113 bacteria not consuming arabinose. Further, the bacteria were grown at different concentrations of the arabinose inducer 0.2, 0.02 and 0.004% and at 37°C for 24 h without or with following incubation at 25°C for 24 h. After induction of expression at 37°C the green fluorescence of NeonOxIrr in all fusion proteins was in average 2.5-fold reduced, compared to the control protein NeonOxIrr (ESI Figure S14a). Further incubation at 25°C did not affect the level of green fluorescence (ESI Figure S14b). The level of the red fluorescence of the mCherry protein was slightly higher for fusion proteins having mCherry at the N-terminus of NeonOxIrr (ESI Figure S14c). The same correlation was maintained with additional incubation at 25°C (ESI Figure S14d). Reduction of the concentration of the arabinose inducer from 0.2% to 0.004% had almost no effect on the level of fusion proteins.

To evaluate the effect of linker length, order of proteins in fusion constructions, and expression conditions on the functional properties of the NeonOxIrr sensor in various fusion proteins, the green fluorescence contrasts after addition of 200 μM H_2O_2 to bacterial suspensions were measured. On average, the contrasts observed in the reaction of the NeonOxIrr sensor with H_2O_2 in bacteria were weakly dependent on the localization of mCherry in fusion proteins and the length of the linkers (ESI Figure S15a). However, in the case of localization of mCherry at the C-end of the NeonOxIrr sensor, a decrease in contrast was observed with a decrease in the length of the linkers. Regardless of the linker length, the maximum achievable contrasts for mCherry localization at the N-end of the sensor were less than for mCherry localization at the C-end. The greatest contrast was observed for fusion protein NeonOxIrr-P2A-mCherry. It practically did not differ from the contrast of the individual sensor NeonOxIrr. Probably, in the process of translation in bacteria, the first protein in the fusion construction is better folded. In the fusion protein tested, the NeonOxIrr sensor on average showed higher contrasts at 0.004% arabinose. Additional incubation at 25°C for 24 h on average resulted in an increase in contrast. A longer additional incubation at 25°C resulted in a decrease in fluorescent contrasts. Thus, the temperature conditions and concentrations of the arabinose inducer were selected for the best expression and reaction with the H_2O_2 for the NeonOxIrr sensor in the fusion protein with mCherry in the bacterial system. When fusion protein was expressed in bacteria, NeonOxIrr-P2A-mCherry showed the greatest contrasts after the addition of H_2O_2. In further experiments on bacteria we decided to use it.

To test the oxidation conditions of the NeonOxIrr-P2A-mCherry ratiometric version in bacteria and compare its functionality with the original NeonOxIrr protein, we varied the H2O2 concentration from 1 to 400 μM. In the range of concentrations of peroxide 10-400 μM, the contrast of NeonOxIrr-P2A-mCherry and NeonOxIrr remained unchanged (ESI Figure S15b). A gradual decrease in contrast was

observed with a decrease in the concentration of H_2O_2 to 1-5 μM, both for NeonOxIrr-P2A-mCherry and for NeonOxIrr (ESI Figure S15c). The contrast drop in the case of NeonOxIrr-P2A-mCherry was observed to a greater extent than for NeonOxIrr. This indicates that the presence of mCherry protein at the C-end of the sensor slightly reduces its sensitivity to low concentrations of hydrogen peroxide. The use of self-cleavable peptide P2A when expressed in animal cells will avoid this limitation.

Development of an alkylation-fixation protocol for bacteria expressing fusion NeonOxIrr-P2A-mCherry using various alkylating agents

To test the alkylation-fixation protocol, two alkylating reagents, IAM and NEM were tested. The influence of various factors on the fluorescence contrast of the fusion sensor after alkylation-fixation was also studied: the concentration of alkylating substances, pH, temperature, treatment with hydrogen peroxide, the concentration of the paraformaldehyde fixing agent.

First, different concentrations of IAM and NEM were tested for the alkylation-fixation protocol in bacteria expressing the fusion sensor NeonOxIrr-P2A-mCherry. The contrast between the oxidized and reduced forms of the sensor without alkylation but after fixing with 4% PFA was reduced about 5-fold, compared to the contrast without alkylation-fixation procedures (ESI Figure S16a-b). When alkylating with either NEM (concentration 40-0.8 mM, pH 7.4) (ESI Figure S16a) or IAM (concentrations 40-0.08 mM, pH 7.4) (ESI Figure S16b), followed by fixation with 4% PFA, the contrast between fusion sensor forms before and after oxidation with 200 μM H_2O_2 was reduced by about 40-60% compared to contrast without alkylation and fixation. In the case of the NEM alkylating agent, concentrations of 0.2 and 0.08 mM did not result in an increase in contrast, as compared to the fixed control without alkylation, probably due to the low alkylation reaction rate.

Storing the fixed samples at 4°C resulted in gradual reduction of the contrast to about 50% of the initial contrast after 48 h of storage, so it is desirable to analyze fresh samples.

For fixation with alkylation by 0.8 mM NEM, the greatest contrast between fusion sensor forms before and after oxidation with H_2O_2 was observed at pH 8.5, and reached about 90% of the contrast without fixation (ESI Figure S16c). At more acidic pH values of 6.5 and 7.4 or more alkaline pH (pH 9.5), we observed a decrease in contrasts. It is known that the alkylation reaction proceeds with the anionic form of the sulfhydryl cysteine group, so the observed gradual decrease in contrast at more acidic pH 6.5 and 7.4 may be due to a decrease in the number of deprotonated sulfhydryl groups of the cysteines of the sensor. Indeed, the sulfhydryl group of cysteine has pK_a ~ 8.2-8.4. Reduction of the contrast with an increase in pH to 9.5 is possibly associated with an increase in the rate of non-specific reaction of NEM with histidine residues, amino groups of lysine residues and N-terminal amino groups.

In the case of fixation with alkylation by 0.2 mM IAM, the greatest contrasts between the oxidized and reduced forms of the fusion sensor were observed in the pH range 6.5-7.4, and were approximately 60-70% of the contrast without fixation (ESI Figure S16d). The alkalinization to pH 8.5 and 9.5 resulted in a gradual decrease in contrast.

To assess the effect of temperature on preserving the contrast after alkylation-fixation, the alkylation of bacteria expressing the fusion sensor NeonOxIrr-P2A-mCherry was carried out at 4, 25 and 37°C. At the alkylation with 0.8 mM NEM, a decrease in temperature to 4°C slightly reduced the contrast after fixation by a factor of 1.6 (ESI Figure S16e), probably due to the slowing down of the alkylation rate with decreasing temperature. In the case of alkylation with 0.2 mM IAM, on the contrary, an increase in temperature to 37 degrees resulted in a significant decrease in the contrast by 5.8-fold, possibly due to an increase in the rate of nonspecific reactions (ESI Figure S16e). Thus, the alkylation with 0.2 mM IAM is equally effective at 4-25°C. Alkylation by NEM with lowering the temperature of the sample to 4°C

requires the use of NEM concentrations greater than 0.8 mM. Indeed, increasing the NEM concentration to 2 mM resulted in a 1.4-fold increase in contrast. Thus, 2 mM NEM is optimal for alkylation at 4°C.

To assess the possibility of blocking endogenous peroxidase activity, fixed samples of bacteria with fusion sensor NeonOxIrr-P2A-mCherry after alkylation-fixation were treated with a 0.3% hydrogen peroxide solution in PBS buffer, pH 7.4 for 30 minutes at room temperature. Treatment with H_2O_2 3- and 17-fold reduced the contrast after alkylation with NEM and IAM, respectively (ESI Figure S17a-b). Treatment with 0.3% H_2O_2 had virtually no effect on green fluorescence and resulted in a significant 2.8 and 3.8-fold quenching of mCherry red fluorescence for samples alkylated with NEM and IAM, respectively. Thus, blocking of endogenous peroxidase activity by treatment with 0.3% H_2O_2 adversely affects both the fusion sensor contrast and the fluorescence level.

To assess the possibility of fixing bacteria with the fusion sensor NeonOxIrr-P2A-mCherry at other concentrations of the fixing agent, samples of bacteria with a fusion sensor after alkylation were treated with 4 or 1% PFA solution. Reduction in the concentration of PFA led to a slight change in the contrast after alkylation with NEM and IAM in 1.6- and 1.7-fold, respectively (ESI Figure S17c-d). Therefore, the proposed alkylation-fixation protocol of the sensor works for different concentrations of the fixing agent.

Thus, optimal conditions for preliminary processing of the sensor with alkylating reagents were chosen, allowing after the fixation to maintain a high contrast of the NeonOxIrr-P2A-mCherry reaction with H_2O_2. Both of the tested alkylating reagents, NEM and IAM, allow maintaining a high contrast of the reaction with H_2O_2 after cell fixation. Optimal conditions for alkylation and fixation were selected. In the case of NEM, alkylation must be carried out at pH 8.5 at concentrations of 0.8 mM or 2 mM at 25 and 4°C, respectively. In the case of IAM, alkylation optimally passes at pH 7.4 at a concentration of 0.2 mM at 4-25°C. Blocking of endogenous peroxidase activity by treatment with 0.3% H_2O_2 is incompatible with the developed alkylation-fixation protocol of the peroxide sensor. The fixation of the fusion sensor after alkylation can be effectively carried out with 1-4% PFA.

Ex vivo H_2O_2 detection failure in neuronal cultures expressing ratiometric NeonOxIrr-P2A-mCherry

We further assessed if the alkylation-fixation conditions optimized for the ratiometric NeonOxIrr-P2A-mCherry expression in HeLa cells would work for neuronal cultures. Neuronal cultures isolated from mouse pups brains were transduced with recombinant viral particles (rAAVs) on day in vitro (DIV) 4th. After incubation with 200 μM external H_2O_2 for 5 minutes, hippocampal and cortical neuronal cultures expressing NeonOxIrr-P2A-mCherry were treated with 1 mM NEM for 10 min followed by fixation with 4% paraformaldehyde for 15-20 min. Then, fluorescence images were acquired for fixed neuronal cultures in green and in red fluorescent channels (ESI Figure S20). After alkylation fixation, we did not observe a significant difference between the green-to-red fluorescence ratios for the H_2O_2-treated and non-treated hippocampal or cortical neuronal cultures, expressing the NeonOxIrr-P2A-mCherry indicator (Figure 6c).

Hence, the alkylation-fixation protocol that distinguished between the H_2O_2-treated and non-treated HeLa cells using the NeonOxIrr-P2A-mCherry peroxide indicator did not work for the neuronal cultures.

Ex vivo H_2O_2 detection failure in the mouse brain expressing ratiometric NeonOxIrr-P2A-mCherry

Because the alkylation-fixation protocol did not work on neuronal cultures, we asked whether this could be related to the different properties of neurons in culture and *in vivo*. This prompted us to apply the alkylation-fixation protocol for the *ex vivo* visualization of external H_2O_2 in the mouse brain. The NeonOxIrr-P2A-mCherry indicator was delivered to both hemispheres of the mouse cerebral cortex via infection with recombinant adeno-associated virus (rAAV) particles. On the 7th day after viral infection, the NeonOxIrr-P2A-mCherry indicator expressed in one of the brain hemispheres was oxidized with an external H_2O_2 injection followed by sequential perfusion of the mouse with the alkylating reagent NEM and PFA. The second brain hemisphere expressing NeonOxIrr-P2A-mCherry construct was injected with a buffer without H_2O_2 to serve as a negative control. When the neocortices were imaged *ex vivo* using confocal microscopy, both green fluorescence of NeonOxIrr and red fluorescence of mCherry were detected in both the cytoplasm and nucleus. The expression of NeonOxIrr has always been co-localized with the expression of mCherry. In addition to the cytoplasm and nucleus, mCherry expression was also observed in the form of granules near the surface of the soma. We did not find significant difference in green-to-red fluorescence ratio in the somas of neurons from hemispheres injected with H_2O_2 or with control buffer (ESI Figure S21). Hence, the alkylation-fixation protocol was not able to preserve the *ex vivo* dynamic range of the NeonOxIrr-P2A-mCherry indicator in response to the external H_2O_2 in the cortex of the mouse brain.

Immunohistochemical (IHC) staining of fixed slices on markers of astrocytes (GFAP) and neuronal cells (NeuN)

To carry out IHC staining of the fixed slices of the brain in which NeonOxIrr-P2A-mCherry was expressed, to neuronal (NeuN) and astrocytes (GFAP)cell markers , staining was performed without prior unmasking. As the primary antibodies, mouse anti-NeuN and rabbit anti-GFAP antibodies were used. As secondary antibodies we applied goat anti-rabbit Alexa 647, goat anti-rabbit DyLight 405 and goat anti-mouse Alexa 647.

In the cerebral cortex, in a region devoid of inflammation, expression of NeonOxIrr-P2A-mCherry was observed in pyramidal neurons of layers 3 and 5, where their morphological characteristics were clearly revealed (ESI Figure S22a). Expression in astrocytes in this region was not observed (ESI Figure S22b). In ESI Figure S22a, a region of the cortex where expression of NeuN and the sensor was present not all neurons expressing the sensor were stained with the NeuN marker. Neurons expressing the sensor in a smaller amount were mostly stained with the NeuN marker. Perhaps in our experiment, the introduced virus or sensor overexpression destroys the phosphorylated isoform of NeuN or interferes with the binding of antibodies.

In places of brain damage, expression of NeonOxIrr-P2A and mCherry was also observed in activated astrocytes with large soma and high expression of GFAP (indicator in ESI Figure S23), as well as in amoeboid-like GFAP-negative cells, most likely cells of activated microglia (arrow in ESI Figure S23).

Thus, it has been shown that the procedure of alkylation-fixation of sections does not affect the epitopes of GFAP and NeuN, and, as a result, the specificity of antibody binding.

Supplementary References

1. Zacharias, D. A.; Violin, J. D.; Newton, A. C.; Tsien, R. Y., Partitioning of lipid-modified monomeric GFPs into membrane microdomains of live cells. *Science* **2002,** 296, (5569), 913-6.
2. Shaner, N. C.; Campbell, R. E.; Steinbach, P. A.; Giepmans, B. N.; Palmer, A. E.; Tsien, R. Y., Improved monomeric red, orange and yellow fluorescent proteins derived from Discosoma sp. red fluorescent protein. *Nat. Biotechnol.* **2004,** 22, (12), 1567-72.
3. Pletneva, N.; Pletnev, V.; Tikhonova, T.; Pakhomov, A. A.; Popov, V.; Martynov, V. I.; Wlodawer, A.; Dauter, Z.; Pletnev, S., Refined crystal structures of red and green fluorescent proteins from the button polyp Zoanthus. *Acta Crystallogr. D Biol. Crystallogr.* **2007,** 63, (Pt 10), 1082-93.
4. Ho, S. N.; Hunt, H. D.; Horton, R. M.; Pullen, J. K.; Pease, L. R., Site-directed mutagenesis by overlap extension using the polymerase chain reaction. *Gene* **1989,** 77, (1), 51-9.
5. Barykina, N. V.; Doronin, D. A.; Subach, O. M.; Sotskov, V. P.; Plusnin, V. V.; Ivleva, O. A.; Gruzdeva, A. M.; Kunitsyna, T. A.; Ivashkina, O. I.; Lazutkin, A. A.; Malyshev, A. Y.; Smirnov, I. V.; Varizhuk, A. M.; Pozmogova, G. E.; Piatkevich, K. D.; Anokhin, K. V.; Enikolopov, G.; Subach, F. V., NTnC-like genetically encoded calcium indicator with a positive and enhanced response and fast kinetics. *Sci Rep* **2018,** 8, (1), 15233.
6. Subach, O. M.; Cranfill, P. J.; Davidson, M. W.; Verkhusha, V. V., An enhanced monomeric blue fluorescent protein with the high chemical stability of the chromophore. *PloS one* **2011,** 6, (12), e28674.
7. Kuzmich, A. I.; Vvedenskii, A. V.; Kopantsev, E. P.; Vinogradova, T. V., [Quantitative comparison of expression for genes linked in bicistronic vectors via ires or 2A-peptide of porcine teschovirus-1 sequence]. *Bioorganicheskaia khimiia* **2013,** 39, (4), 454-65.
8. Subach, F. V.; Patterson, G. H.; Manley, S.; Gillette, J. M.; Lippincott-Schwartz, J.; Verkhusha, V. V., Photoactivatable mCherry for high-resolution two-color fluorescence microscopy. *Nat. Methods* **2009,** 6, (2), 153-9.
9. Shaner, N. C.; Steinbach, P. A.; Tsien, R. Y., A guide to choosing fluorescent proteins. *Nat Methods* **2005,** 2, (12), 905-9.
